# Kharon Is Crucial for *Trypanosoma cruzi* Morphology but Does Not Impair In Vitro Infection

**DOI:** 10.3390/pathogens14040312

**Published:** 2025-03-25

**Authors:** Jose Luis Saenz-Garcia, Normanda Souza-Melo, Juliana Severo Miranda, Beatriz Borges, Lisandro A. Pacheco-Lugo, Jose M. Quintero-Solano, Nilmar Moretti, Richard Wheeler, Lia C. Soares-Medeiros, Wanderson D. DaRocha

**Affiliations:** 1 Laboratório de Genômica Funcional de Parasitos (GFP), Universidade Federal de Paraná, Curitiba 81531-980, Brazil; 2 Laboratório de Ultraestrutura Hertha Mayer, Universidade Federal do Rio de Janeiro (UFRJ), Rio de Janeiro 21491-590, Brazil; normandasouzamelo@gmail.com; 3 Laboratório de Biologia Molecular de Patógenos (LBMP), Departamento de Microbiologia, Imunologia e Parasitologia, Escola Paulista de Medicina, Universidade Federal de São Paulo, São Paulo 04023-062, Brazil; 4 Laboratório de Biologia Celular, Instituto Carlos Chagas, Fundação Oswaldo Cruz (Fiocruz), Curitiba 81310-020, Brazil; 5 Facultad de Ciencias Básicas y Biomédicas, Universidad Simón Bolívar, Barranquilla 080020, Colombia; 6 Laboratorio de Biotecnología Farmacéutica, Centro de Biotecnología Genόmica, Instituto Politécnico Nacional, Reynosa 88710, Mexico; 7 Nuffield Department of Medicine, University of Oxford, Oxford OX1 3SY, UK

**Keywords:** *Trypanosoma cruzi*, morphology, CRISPR/Cas9

## Abstract

Chagas disease, caused by *Trypanosoma cruzi*, is a neglected tropical disease with few options for treatment and no available vaccine. Deletion mutants for live attenuated vaccines, particularly deletions of proteins related to the cytoskeleton, have been widely tested in related parasites but candidates have not been tested in *T. cruzi*. Kharon is one such protein, identified as being associated with the cytoskeleton in *Leishmania* and essential for amastigote replication. Here we investigated the *T. cruzi* Kharon ortholog (*Tc*Kharon) to test if it has orthologous function and thus potential in generating a live attenuated vaccine. In silico analysis predicted *Tc*Kharon to be an intrinsically disordered protein, consistent with its ortholog feature, and GFP fusion protein revealed that *Tc*Kharon is associated with the cytoskeleton of epimastigotes. CRISPR-Cas9-mediated gene disruption impaired epimastigote proliferation and cytokinesis, resulting in altered nucleus-to-kinetoplast ratios and pronounced morphological defects, particularly in the posterior cell region. Despite these abnormalities, *Tc*Kharon^−/−^ mutants retained the ability to differentiate into metacyclic trypomastigotes and exhibited in vitro infection rates comparable to wild-type parasites. Our data show that *Tc*Kharon is crucial for cell morphology. However, in contrast to close related parasites, *Tc*Kharon is not essential for in vitro infectivity.

## 1. Introduction

Chagas disease is a neglected tropical disease that affects 6–8 million people in Latin America [1]. Available treatments for the chronic stage of the disease have limited efficiency and significant side effects [2], while the development of vaccines is hindered by the fact that the causative agent, *Trypanosoma cruzi*, uses specialized mechanisms of immune escape. Thus far, few *T. cruzi* vaccine candidates have been investigated, yielding unsatisfactory results [3]. The use of live attenuated vaccines appears to be an option for diseases caused by trypanosomatids since it has been shown that inoculation with a living avirulent strain of a parasite protects against challenge with a respective pathogenic strain [4,5,6].

Currently, no approved vaccine exists for Chagas disease. Among the various vaccination strategies, live attenuated vaccines offer a promising alternative, although their development presents significant challenges. While some potential targets have been identified [7,8], and tested vaccines have reduced disease burden, the risk of relapse remains. An effective target for attenuation must prevent vector-borne parasite transmission, ensure stable attenuation (e.g., through gene knockout), exhibit limited replication to ensure safety, and elicit a robust immune response. Identifying novel targets for generating attenuated parasites is therefore crucial. Genes encoding proteins involved in replication, metabolism, and cell cycle progression are considered promising candidates [9,10]. Certain cytoskeleton-associated proteins, which play roles in cell division, motility, cytokinesis/mitosis, and organelle positioning, may fulfill these criteria [6,9,10].

Microtubules, which are key components of the cytoskeleton, are found in the flagellar axoneme, basal body, and subpellicular microtubules of *T. cruzi*. This last structure, located near the plasma membrane, maintains the parasite’s shape [11] and likely contributes to its survival in both insect and mammalian hosts. The subpellicular microtubules form a crosslinked network through microtubule-associated proteins (MAPs) [11]. Studies of *T. brucei* have identified over 70 proteins associated with the subpellicular array [12], with some localized throughout the array and others to specific subdomains, such as the cell body, reflecting potential local specializations (reviewed by Sinclair et al. [13]).

Subpellicular array-associated proteins are of interest as the deletion of genes encoding proteins that impair *Leishmania* division in the mammalian host. Notably, centrin-4 and Kharon-1 have been identified as candidates for parasite attenuation in vaccine development [6,14,15]. Interestingly, Kharon-1 was first identified in *L. mexicana* as a protein necessary for the specific localization of glucose transporter-1 (*Lmx*GT1) to the flagellar membrane [16]. Similar function was later shown in *T. brucei* [17]. Endogenous tagging showed that Kharon localizes to the subpellicular microtubules, the basal body in *L. mexicana* [18], at the base of the flagellum, or subpellicular microtubules and mitotic spindle in bloodstream forms of *T. brucei* [17]. RNAi knockdown of *Tb*Kharon showed that it is necessary for normal cell division of the procyclic and bloodstream forms [17] while a deletion mutant showed that *Lmx*Kharon is necessary for normal cell division, but only in the amastigote form [18]. This mammalian stage-specific phenotype led to its gaining particular interest as a live attenuated vaccine candidate.

Kharon is one of a set of proteins that are spatially proximal, localized to the entire subpellicular microtubule array, and are necessary for normal division and morphogenesis. Two *Lm*Kharon-associated proteins, which localize only to the subpellicular microtubules, were identified by proximity labeling (BioID) [15]. The first (KHAP1, an ortholog of *T. brucei* MARP1) is necessary for normal *Leishmania* division [15], as would be expected given the *T. brucei* RNAi phenotype. MARP-1was previously identified as a repetitive-domain contained protein that binds to microtubules of the cytoskeleton in *T. brucei* [19]. The second (KHAP2) was also necessary for normal division [15]. In *T. brucei* CAP5.5 proximity labeling independently identified the KHAP2 *T. brucei* ortholog (which was named CAP50), along with CAP42 and CAP52, all of which were necessary for normal morphogenesis and cytokinesis [20]. Kharon is therefore one of a set of closely positioned subpellicular-associated proteins that are necessary for normal morphogenesis and cytokinesis (MARP, CAP5.5, Kharon, KHAP2, CAP52 and CAP42), but itself has an additional function in defining specialized plasma membrane domains.

While these and other subpellicular array-associated proteins have orthologs in many trypanosomatids, they often have high sequence divergence and do not necessarily have the same function. This can be illustrated with a protein in a different cytoskeletal structure. For example, FLA-1 in the flagellum attachment zone is essential for cytokinesis in *T. brucei*, however, its ortholog gp72 in *T. cruzi* can be deleted [21,22]. This highlights the need for studying these molecular players in each species.

Here, we analyze the cellular phenotype resulting from genetic disruption of the *T. cruzi* Kharon ortholog (*Tc*Kharon). Our aims were to: (1) determine *Tc*Kharon’s role in cell morphogenesis and division across different life stages, and (2) assess its potential as a target for parasite attenuation in vaccine development. Using markerless CRISPR-Cas9-mediated *Tc*Kharon disruption, we demonstrate that *Tc*Kharon is essential for normal morphogenesis but not for in vitro host cell infection.

## 2. Material and Methods

### 2.1. Bioinformatic Analysis

Kharon amino acid sequences from *T. cruzi* and related Trypanosomatids were retrieved from TriTrypDB (https://tritrypdb.org/tritrypdb/app accessed on 26 March 2021). The sequences were aligned using Clustal Omega and a phylogenetic tree was built in the SeaView v. 5.0.4 software [23]. pLDDT prediction and 3D structure prediction were performed using “ColabFold” (https://colab.research.google.com accessed on 18 May 2021). The colors in the 3D structure indicate the pLDDT values of each residue.

### 2.2. Parasite Maintenance and Growth Curve

*T. cruzi* strain Dm28c and LLC-MK2 cells were obtained from FIOCRUZ-PR (Carlos Chagas Institute—Curitiba/Brazil). *T*. *cruzi* epimastigotes of the Dm28c clone were cultured in a liver infusion tryptose (LIT) medium supplemented with heat-inactivated fetal bovine serum (FBS; 10%), hemin, and penicillin/streptomycin [24]. Metacyclic trypomastigotes (MTs) were obtained using the protocol described by Contreras et al. [25]. Briefly, epimastigotes were induced to MT differentiation by incubation on TAU media, and the MTs were purified on a DEAE-Column, and quantified. For growth curve analysis, epimastigotes were counted daily in a Neubauer chamber, and when the culture reached the stationary phase, the culture was diluted in 1× phosphate buffered saline (PBS).

### 2.3. Molecular Cloning and In Vitro Transcription

For the generation of Kharon-null mutants (*Tc*Kharon^−/−^), first a single guide RNA (sgRNA) was designed and cloned as described by Saenz-Garcia et al. [26]. Briefly, the EuPaGDT v1.0 software (http://grna.ctegd.uga.edu/ accessed on 15 February 2018) was used to find sgRNA target sequences in the *Tc*Kharon coding sequence (ID = C4B63_14g70) from the Dm28c genome (TriTrypDB-28) considering the protospacer motif sequence (PAM sequence) of *Sa*Cas9 nuclease [27]. From the list that was returned, a sgRNA was selected by considering the score, position (located in the first half of the coding sequence), and whether the sgRNA target sequence was conserved among almost all available *T. cruzi* genomes.

The oligonucleotides corresponding to the target sequence (sgRNA-*Tc*Kharon608-Plus ATAGGCGCTTCCATGAACAAACCAGC and sgRNA-*Tc*Kharon608-Minus AAACGCTGGTTTGTTCATGGAAGCGC) were annealed and cloned into the pT7-*Sa*Cas9sgRNA-BsaI plasmid [26] that had been previously digested with *Bsa*I, thus generating pT7-*Sa*Cas9-sgRNA-*Tc*Kharon608. This plasmid was used as a template for in vitro transcription, as suggested by the manufacturer’s protocol (MEGAscript™ T7 Transcription Kit—Thermo Fischer Scientific, Waltham, MA, USA).

To recover *Tc*Kharon expression in *Tc*Kharon^−/−^ parasites or for overexpression in wild-type cells, the *Tc*Kharon coding sequence was fused to the GFP N-terminus, as follows. The primers, *Tc*Kharon-For-*Xba*I (5′-TCATCTAGAATGGCCACGGCAGCAGTTGAGC-3′) and *Tc*Kharon-Rev-*Bgl*II (5′-TTTAGATCTGGAAGATGTTGTTGATGCCGGTT-3′) were used for PCR amplification using genomic DNA from Dm28c clone. The PCR product was digested with *Xba*I/*Bgl*II, and cloned in the pTREX-Amastin::GFP-Neo plasmid [28] that had been previously digested with *Xba*I/*Bam*HI. The new plasmid, named as pTREX-*Tc*Kharon::GFP-Neo, was transfected by electroporation in the wild-type (WT) and *Tc*Kharon^−/−^ epimastigote cells, as previously described by Pacheco-Lugo et al. [29].

### 2.4. CRISPR/Cas9 Editing and Genotyping

Genome editing using CRISPR/Cas9 to disrupt *Tc*Kharon and genotyping of *Tc*Kharon^−/−^ mutants were performed as described by Saenz-Garcia et al. [26]. Briefly, epimastigotes were transfected once or twice using the ribonucleoprotein (RNP) complex (*Sa*Cas9 plus *Tc*Kharon-specific sgRNA) in the presence of donor ssDNA (synthetic oligonucleotide) containing a sequence of three stop codons at different open reading frames to ensure coding sequence disruption after DNA repair and a *Bgl*II restriction site flanked by 25 nucleotides upstream and downstream of the *Sa*Cas9 cleavage site (AAAGCGCCGCTTCCATGAACAAACCTGAATGACTG*A**GATCT*AAGCGTGAATCTCTTTGGACGTACAG). The parasites electroporated with the RNP and donor sequence were tested for gene disruption by PCR followed by digestion with the *Bgl*II enzyme.

### 2.5. Scanning and Transmission Electron Microscopy

For scanning electron microscopy (SEM) analyses, the parasite samples were processed as described by Souza-Melo et al. [30]. Briefly, epimastigotes were fixed in 2.5% glutaraldehyde diluted in cacodylate buffer (0.1 M, pH 7.2) for 1 h. The cells were adhered to poly-L-lysine coverslips, and treated with 1% osmium tetroxide diluted in cacodylate buffer for 1 h. Samples were dehydrated in a graded ethanol series (50%, 70%, 90%, and two exchanges of 100% ethanol for 10 min each step), and critical-point dried using CO_2_. The slides were coated with a 5 nm layer of platinum and then visualized in an EVO 10 and ZEISS FIB-SEM AURIGA 40 (Carl Zeiss Microscopy GmbH—Oberkochen, Baden-Württemberg, Germany) scanning electron microscope at the National Center for Structural Biology and Bioimaging (CENABIO) at UFRJ. Cell lengths were measured in the SEM images using the AxioVision4 program.

For transmission electron microscopy (TEM), log-phase epimastigotes were treated following the procedure described for scanning microscopy [31]. After treatment, the cells were washed with cacodylate buffer (0.1 M, pH 7.2) and fixed in cacodylate buffer (0.1 M, pH 7.2) containing paraformaldehyde (4%) and glutaraldehyde (2.5%). All samples were postfixed in osmium tetroxide (1%), dehydrated with acetone series, and embedded in EMbed 812^®^ resin (Electron Microscopy Sciences—Hatfield, PA, USA.). Grids containing ultrathin sections (70 nm) were stained with uranyl acetate and lead citrate, and observed under a transmission electron microscope (TEM; JEOL JEM-1400Plus—JEOL Ltd., Akishima, Tokyo, Japan) at Carlos Chagas Institute (FIOCRUZ-PR, Curitiba, PR, Brazil).

### 2.6. Immunofluorescence Assay and Localization of GFP-Tagged TcKharon

Epimastigotes of WT, *Tc*Kharon^−/−^, and the addback cell lines, *Tc*Kharon-AB (*Tc*Kharon^−/−^ carrying pTREX *Tc*Kharon::GFP) were fixed with 4% paraformaldehyde and coated on polylysine coverslips for 20 min. Coverslips were washed with PBS and the parasites were permeabilized with PBS containing 0.1% Triton X-100 and then blocked with 3% (*m/v*) bovine serum albumin (BSA) for 1 h at room temperature. The samples were incubated for 16 h at 4 °C with the monoclonal antibody 2F6 (mAb 2F6, 1:100) and the polyclonal anti-α-tubulin antibody (1:200), which recognize a flagellar protein of ~70 kDa [32] and α-tubulin polymers, respectively. The samples were washed three times with PBS containing 0.05% Tween-20, and incubated with the secondary antibody (anti-mouse conjugated with Alexa-488—Thermo Fisher Scientific, Waltham, MA, USA), 1:500. The coverslips were washed three times, mounted on a microscope slide, and analyzed by confocal microscopy (Nikon A1R Multiphoton confocal microscope—Nikon Corporation, Tokyo, Japan). Parasites of *Tc*Kharon-AB, WT overexpressing *Tc*Kharon::GFP, and WT overexpressing GFP alone (pTREX-GFP) [33] were also analyzed for confocal microscopy as detailed above. Cytoskeletons from these parasites were obtained by incubating the parasites with 40 μL of cold PEME (100 mM PIPES, 1 mM MgSO_4_, 0.1 mM EDTA, 2 mM EGTA, pH 6.9) containing Triton X-100 (1% *v/v*), and then fixed. The images from confocal microscopy were analyzed with the Fiji 2.3.0 (Image J2) software.

### 2.7. Western Blot

Epimastigotes (5 × 10^7^ cells) of *Tc*Kharon-AB and WT expressing *Tc*Kharon::GFP were harvested, washed once with PBS, resuspended in Laemmli buffer, sonicated for 3 min, then electrophoresed on a 12% SDS-PAGE gel. The proteins were transferred to a nitrocellulose membrane, then the membrane was washed with PBS 1× and blocked with 5% non-fat milk in PBS. The membranes were incubated with a polyclonal anti-GFP antibody (1:1000), washed, and then incubated with a secondary anti-rabbit antibody conjugated with peroxidase (1:1000). The antibody recognition was detected using the ECL Chemiluminescence Kit (Thermo Fisher Scientific, Waltham, MA, USA), and revealed on an X-ray film. Images were acquired by exposing X-ray films on the UVP Bioimaging system.

### 2.8. Cell Infection Assays and Tissue-Cultured Derived Trypomastigote Counts

LLC-MK2 monolayers cultured to 100% cell confluence were treated with trypsin (0.05%) (Thermo Fisher Scientific, Waltham, MA, USA) and washed twice with PBS. Cells (4 × 10^4^ cells/well) were placed in a 24-well plate containing coverslips and cultured in RPMI 1640 (Thermo Fisher) supplemented with 5% FBS in 5% CO_2_ at 37 °C. Tissue culture-derived trypomastigotes (TCTs) of WT, *Tc*Kharon^−/−^, and *Tc*Kharon-AB obtained from a previous infection of LLC-MK2 cells with metacyclic trypomastigotes were used at a ratio of 10 parasites per LLC-MK2 cell (MOI of 10:1) for 5 h. After the infection period, cells were washed with PBS three times to remove extracellular forms, and fresh RPMI media supplemented with 5% FBS was added. The samples were washed, fixed, and analyzed on an Operetta CLS High Content Analysis System (PerkinElmer, Waltham, MA, USA).

### 2.9. Statistical Analysis

Each experiment was conducted in triplicate before analysis. A paired *t*-test was employed to compare two groups. One-way ANOVA with Tukey’s post-hoc test was employed for the statistical analysis among several groups. A *p*-value lower than 0.05 was considered statistically significant. GraphPad version 8.0.2 was used for statistical analysis and graphs.

## 3. Results

### 3.1. Kharon Is an Intrinsically Disordered Protein Based on the 3D Structure Prediction

**Figure 1:**
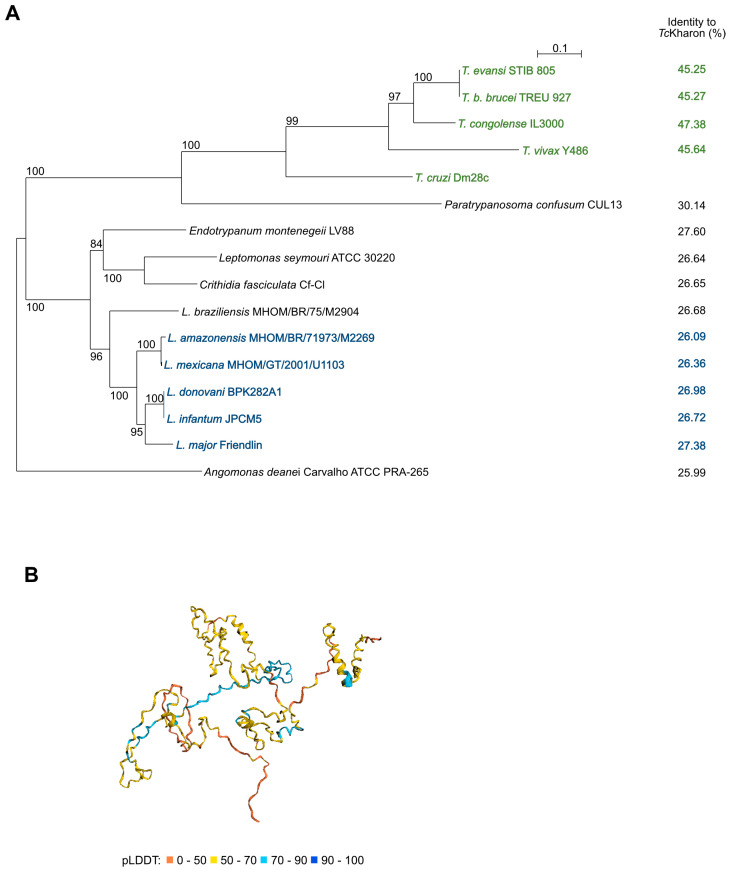
Phylogenetic tree from protein sequences of related trypanosomatids, and *Tc*Kharon structure prediction using AlphaFold2. (**A**) Amino acid sequences from TriTrypDB (https://tritrypdb.org/tritrypdb/app accessed on 26 March 2021) were aligned using the SeaView software. The aligned sequences were used to generate a phylogenetic tree (SeaView 5.4). (**B**) Prediction model from https://colab.research.google.com/github/sokrypton/ColabFold/blob/main/AlphaFold2 (accessed on 26 March 2021). The colors indicate the predicted pLDDT values for *Tc*Kharon as given in the key.

The multiple sequence alignment of Kharon using Clustal Omega showed low conservation among the Trypanosomatids, which included *T. cruzi*, *Trypanosoma congolense*, *Trypanosoma vivax*, *T. b. brucei*, *Paratrypanosoma. confusum*, *Endotrypanum monterogeii*, *Leishmania major*, *Leishmania donovani*, *Leishmania infantum*, *Crithidia fasciculata*, *Leptomonas seymouri*, *Leishmania mexicana*, *Leishmania amazonensis*, *Angomonas deanei*, and *Leishmania braziliensis*, as indicated in Supplementary Figure S1. A phylogenetic tree showed that Kharon sequence similarity mirrors the known species phylogeny (Figure 1A). The highest identity to *Tc*Kharon was the ortholog from *T. congolense* (47.38% identity), while the lowest was the ortholog from *A. deanei* (25.99% identity). Notably, we were unable to identify Kharon orthologs in *Bodo saltans* or outside the kinetoplastid lineage.

Recently, DeepMind developed AlphaFold, marking a significant advancement in predicting protein tertiary structures from primary sequences [34]. In collaboration with EMBL-EBI, AlphaFold was used to model the structure of a curated subset of UniProt, including *Tc*Kharon (accession V5B3K7). The *Tc*Kharon prediction yielded a low predicted local distance difference test (pLDDT) score, which is often associated with intrinsically disordered regions (IDRs) [35]. However, AlphaFold generates its most confident predictions from deep multiple sequence alignments, and the DeepMind/EMBL-EBI pipeline is not well-optimized for divergent lineages that are underrepresented in UniProt, such as trypanosomatids [36]. Therefore, low pLDDT scores may reflect genuine structural uncertainty rather than IDRs. To improve accuracy, a version of ColabFold [37] optimized for trypanosomatids [36] was used for structure prediction. The resulting models for *Lmx*, *Tb*, and *Tc*Kharon exhibited similar structural features, including consistently low pLDDT scores (see Supplementary Figure S2). Furthermore, PONDR, a traditional IDR prediction tool indicates that the overall disordered residues range from 48.65 to 74.32%, aligning with high proline content (>10%), and charged amino acids (>28.5%) observed in *Lmx*, *Tb* and *Tc*Kharon (see Supplementary Figure S3).

### 3.2. TcKharon::GFP Is Localized at the Subpellicular Cytoskeleton

**Figure 2:**
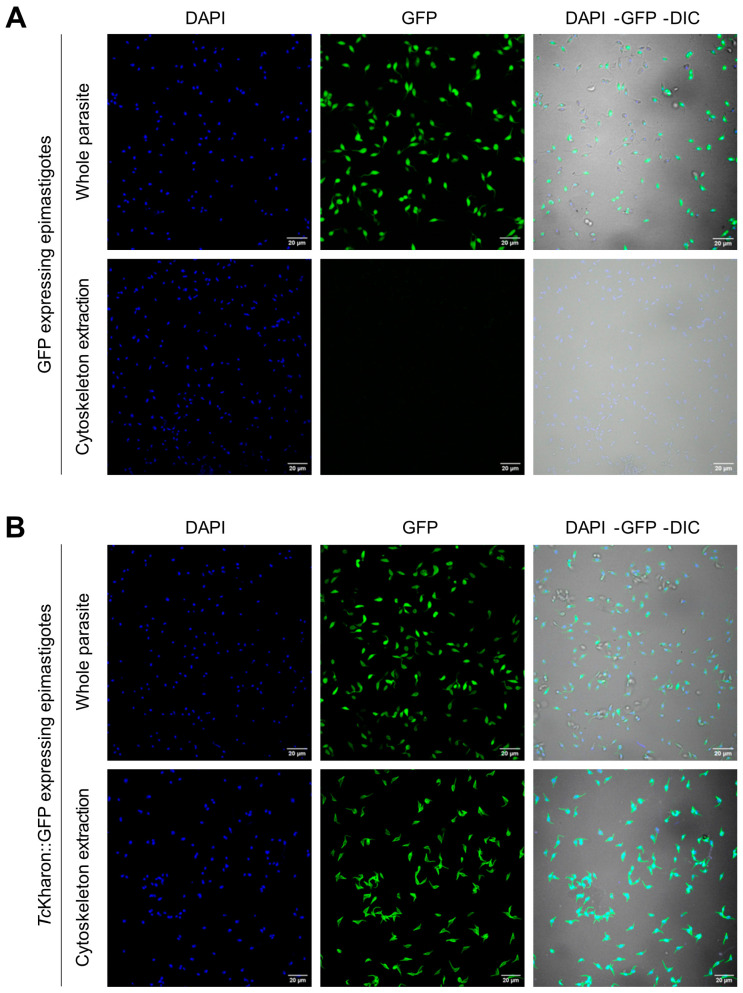
Localization of *Tc*Kharon tagged with GFP. WT parasites transfected with the plasmid pTREX-GFP (control plasmid—epimastigotes expressing GFP) (**A**), or pTREX-*Tc*Kharon::GFP (epimastigotes expressing *Tc*Kharon::GFP) (**B**). These cells were either treated or not treated with PEME buffer containing TritonX-100 for cytoskeleton extraction as indicated (see Section 2). Left images, DAPI staining (blue); center images, GFP fluorescence (green); and right images, overlay of DAPI, GFP fluorescence, and DIC images.

To determine the cellular localization of *Tc*Kharon and whether it is associated with the *T. cruzi* cytoskeleton, Dm28c WT epimastigotes were transfected with pTREX-GFP or pTREX-*Tc*Kharon::GFP. Parasites overexpressing GFP or *Tc*Kharon::GFP were subjected to confocal microscopy analysis (Figure 2A,B), wherein both parasites showed GFP fluorescence throughout the whole cell body. When the epimastigotes were treated with detergent for cytoskeleton extraction, as previously described [38], GFP signal was lost (Figure 2B), while *Tc*Kharon::GFP retained fluorescence signal in the detergent-insoluble cortical cytoskeleton. This distribution seems similar to the distribution of *Lmx*Kharon or *Tb*Kharon to the subpellicular microtubules [17,18].

### 3.3. TcKharon Disruption Affects Cell Growth and Cytokinesis of Epimastigotes

**Figure 3:**
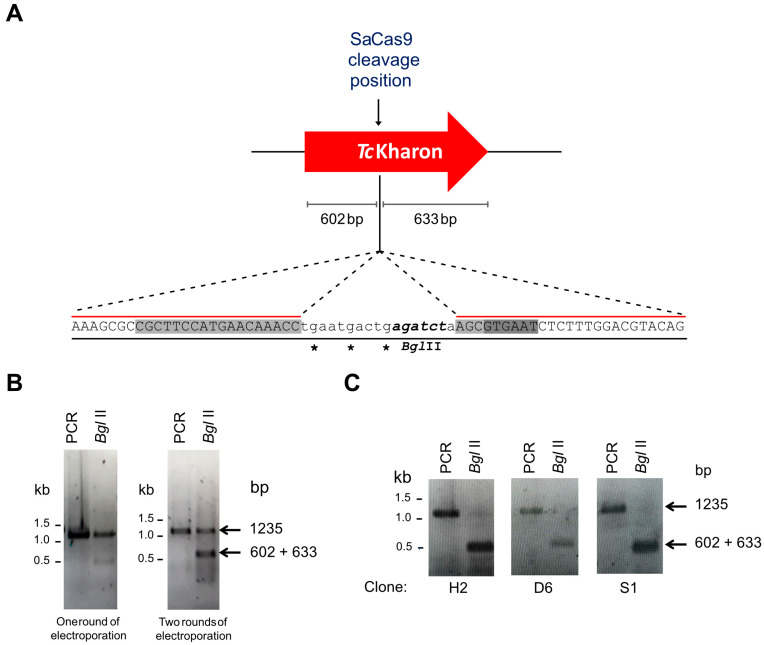
Genome editing of *Tc*Kharon using CRISPR/Cas9. (**A**) Schematic representation of the sgRNA targeting site by the *Sa*Cas9 ribonucleoprotein (RNP) complex in the *Tc*Kharon gene (C4B63_14g70). The RNP complex cleaves right after nucleotide 585 of the *Tc*Kharon coding sequence (CDS length: 1218 bp). The insertion point of a *Bgl*II restriction site (italics) is represented in order to easily track parasite editing through DNA digestion. Stop codons (asterisks) ensure CDS disruption by homologous recombination using a donor DNA sequence. The sequence highlighted in light gray in the donor sequence corresponds to the sgRNA target site, and the dark gray sequence is the PAM sequence. (**B**) Genotyping of *Tc*Kharon showing genome editing of wild-type (WT) parasites. The gels show undigested PCR product (amplicon sizes: WT *Tc*Kharon = 1218 bp and *Tc*Kharon^−/−^ = 1235 bp) and *Bgl*II-digested (*Bgl*II) PCR product of the full-length open reading frame (ORF) of *Tc*Kharon of two cultures. The left image corresponds to the PCR product and *Bgl*II-digested PCR derived from a mixed population of parasites transfected once with *Sa*Cas9 RNP plus donor sequence. The right gel corresponds to the genotyping of a culture transfected twice with RNP complex plus donor DNA. (**C**) Genotyping of individual clones containing both *Tc*Kharon edited alleles.

To analyze *Tc*Kharon function, genome editing with the CRISPR/Cas9 system was applied to the central part of the *Tc*Kharon gene, as shown in Figure 3A. Similar to our previous work [26], two rounds of electroporation were required to improve genome editing, since the *Tc*Kharon PCR product of the edited population is more sensitive to *Bgl*II digestion (since this restriction site is present on the oligonucleotide donor) compared to one round of transfection (Figure 3B). For identification of homozygous edited clones, limiting dilutions were carried out followed by genotyping. Both alleles were edited in three out of 12 of the clones, as detected by *Bgl*II digestion that produced the two expected fragments of 602 and 633 bp, which were not resolved in these electrophoresis conditions (Figure 3C). The efficiency in generating homozygous clones was also similar to that observed in our previous work [26].

**Figure 4:**
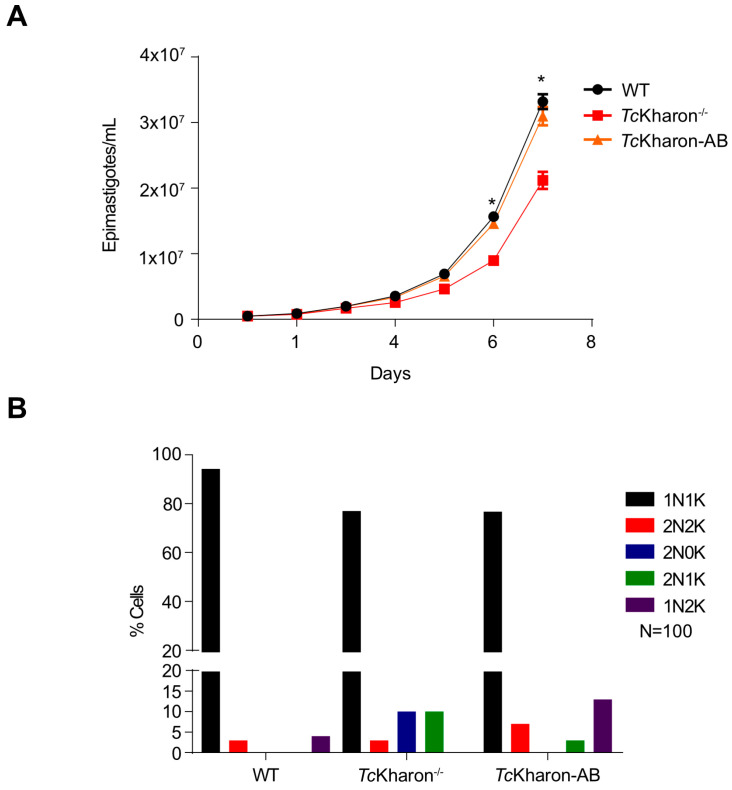
Growth of *Tc*Kharon^−/−^ is impaired. (**A**) Parasites were cultured for 7 days in LIT medium and counted daily. Asterisks indicate statistically significant differences between WT and *Tc*Kharon^−/−^ at *p <* 0.05. TcKharon-AB corresponds to *Tc*Kharon^−/−^ overexpressing *Tc*Kharon::GFP. These experiments were performed in triplicate. (**B**) Quantification of nuclei and kinetoplast numbers through DAPI staining of cells.

The disruption of *Tc*Kharon affected the cellular multiplication of epimastigotes in vitro. The number of parasites was markedly reduced from day 5 to 7 when compared to the WT cells. At day 6, WT cells achieved a number of 1.5 × 10^7^ cells/mL ± 5.8 × 10^5^ while *Tc*Kharon^−/−^ reached 8.9 × 10^6^ cells/mL ± 1.73 × 10^5^. As expected, adding back *Tc*Kharon in the deficient line (*Tc*Kharon-AB) recovered the epimastigote growth fitness, reaching 1.25 × 10^7^ cells/mL ± 5.66 × 10^5^ (Figure 4A).

To evaluate abnormalities in cell division affecting the number of nuclei (N) and kinetoplasts (K) per cell, WT and the mutant clone were DAPI stained and the DNA positive organelles were counted. After counting at least 100 cells of a log phase population, some alterations in the percentage of the N:K cell types were observed (Figure 4B, and Supplementary Figure S4). For WT, 93% of cells were 1N1K, 3% were 1N2K, and 4% were 2N2K. On the other hand, 77% of the *Tc*Kharon^−/−^ cells were 1N1K, 3% were 2N2K, 10% were 2N1K, and 10% were 2N0K. The data show that *Tc*Kharon disruption affected epimastigote division. Despite completely recovering the epimastigote cell growth, the *Tc*Kharon-AB cell population only partially recovered the percentage N:K phenotype of WT cells (Figure 4B). This may represent a lower rate of aberrant division, and persistence of the aberrant cells in the population.

### 3.4. Morphology of TcKharon-Null Epimastigotes Strongly Affects the Posterior Cell Region

**Figure 5:**
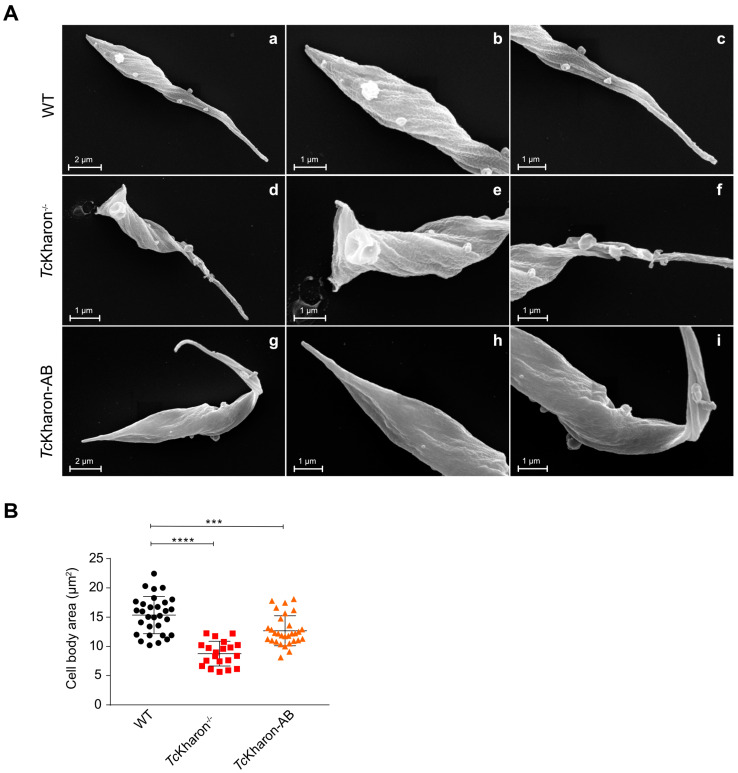
*Tc*Kharon^−/−^ epimastigotes are smaller in size. (**A**) SEM images of *T. cruzi* WT (**a**–**c**), *Tc*Kharon^−/−^ (**d**–**f**), and addback (*Tc*Kharon-AB) (**g**–**i**) cell lines. Central (**b**,**e**,**h**), and right (**c**,**f**,**i**) images correspond to zoom of both ends of the parasites. (**B**) Scatter plot of cell body area**.** At least 25 cells were analyzed using Fiji 2.3.0 software (N = 25). Asterisks represent statistically significant differences between groups at *** *p* < 0.001 and **** *p* < 0.0001.

The *Tc*Kharon^−/−^ mutant exhibited a distinct morphological abnormality, with most epimastigotes adopting a rounded shape that deviated significantly from the typical *T. cruzi* epimastigote form (Figure 5A). Images acquired from SEM and TEM of the epimastigotes enabled analysis of the cell body area, wherein the WT parasites had an average area of 15.36 ± 3.19 µm^2^, *Tc*Kharon^−/−^ parasites were 8.77 ± 2.19 µm^2^, and *Tc*Kharon-AB parasites were 12.68 ± 2.87 µm^2^ (Figure 5B). The difference in cell body area between WT and *Tc*Kharon^−/−^ parasites was statistically significant (*p* < 0.0005), and although *Tc*Kharon-AB appeared to have a greater cell body size than *Tc*Kharon^−/−^, this was still significantly smaller than WT (*p* < 0.005).

**Figure 6:**
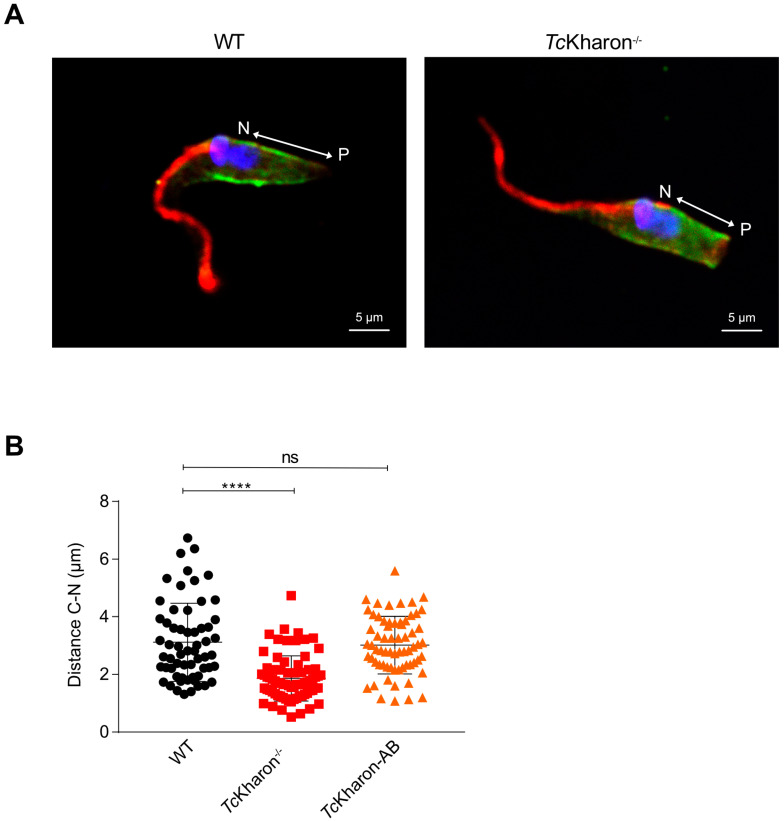
The posterior end of *Tc*Kharon^−/−^ epimastigotes is shortened. (**A**) Immunofluorescence of *Tc*Kharon^−/−^ and WT cells stained with DAPI (blue) and the antibodies to 2F7 (flagellum; red) and α-tubulin (green). (**B**) Scatter plot showing data from the measures of the distance between the N (Nucleus) and P (Posterior Cortical Tip). The asterisk represents a statistically significant difference at **** *p* < 0.0001; ns represents a non-statistical difference.

Next, the distance between the nucleus (N) and posterior cell tip (P) was analyzed in cells stained with DAPI and the α-tubulin antibody (Figure 6A). The distance between N and P in WT epimastigotes was 3.14 ± 1.36 µm, in *Tc*Kharon^−/−^ mutants the distance was 1.86 ± 0.78 µm, and 3.05 ± 0.99 µm for *Tc*Kharon-AB cells. The N–P distance was found to be statistically different when comparing WT to *Tc*Kharon^−/−^ cells (*p* < 0.0005), but not with *Tc*Kharon-AB cells (Figure 6B). This indicates that *Tc*Kharon disruption impacts parasite morphology, and this can be complemented by reintroducing *Tc*Kharon expression.

### 3.5. Metacyclogenesis and Cell Invasion of the TcKharon^−/−^ Mutant Are Not Affected

**Figure 7:**
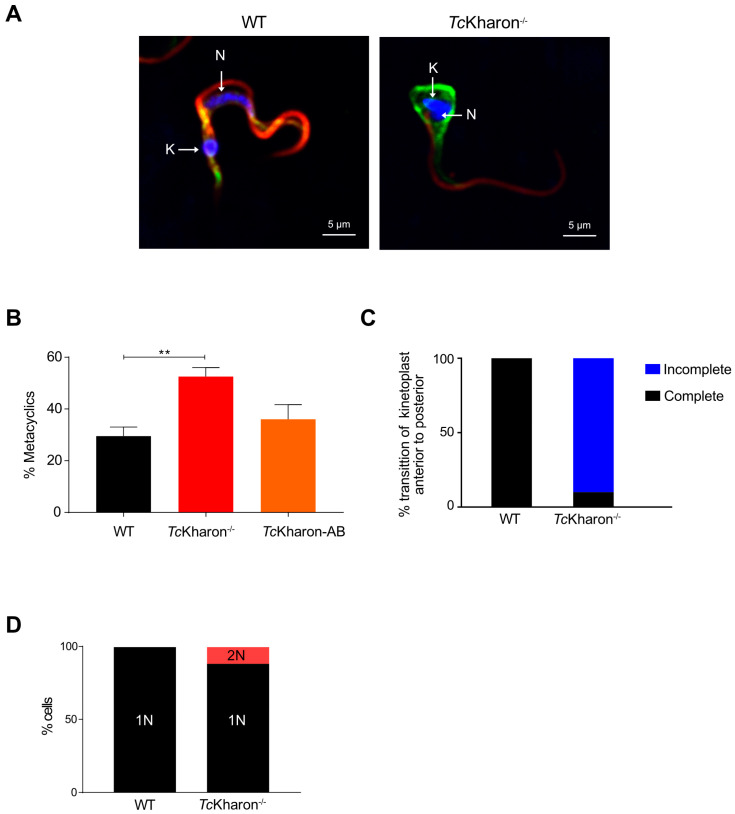
Metacyclogenesis of *Tc*Kharon^−/−^ mutants. (**A**) Immunofluorescence of metacyclic trypomastigotes (MTs) from WT and *Tc*Kharon^−/−^ cultures showing the defect in morphology. Antibodies to 2F7 (flagellum; red) and actin (green) were used. White arrows indicate the nucleus and kinetoplast. (**B**) Percentage of metacyclic cells. Asterisk indicates statistically significant difference at *p* < 0.05. (**C**) Bar graphs showing the transition of the kinetoplast from the anterior to posterior region of the cell body. (**D**) Bar graphs showing the number of binucleated cells in the total cell population.

Epimastigotes were induced to differentiate into metacyclic trypomastigotes (MTs) as described by Contreras [25]. Since *Tc*Kharon^−/−^ presented abnormal epimastigotes (Figure 5A), the MTs were separated from epimastigotes by chromatography using the DEAE-Cellulose column [39], and the MTs yield determined by cell counting. The purified forms from null mutants (i.e., probable MTs) showed morphological alterations (Figure 7A) The *Tc*Kharon^−/−^ mutant showed a higher percentage of metacyclic cells (48.50 ± 2.12%) compared to *Tc*Kharon-AB (36.00 ± 5.65%) and WT (29.50 ± 3.53%) (Figure 7B). The positive identification of metacyclic cells was challenging as almost 90% of the cells in the *Tc*Kharon ^−/−^ cell line did not exhibit a clear kinetoplast transition from the anterior to the posterior region of the cell (Figure 7C). Besides cell morphology alterations, 12% of binucleated cells were observed in null mutants (Figure 7D).

**Figure 8:**
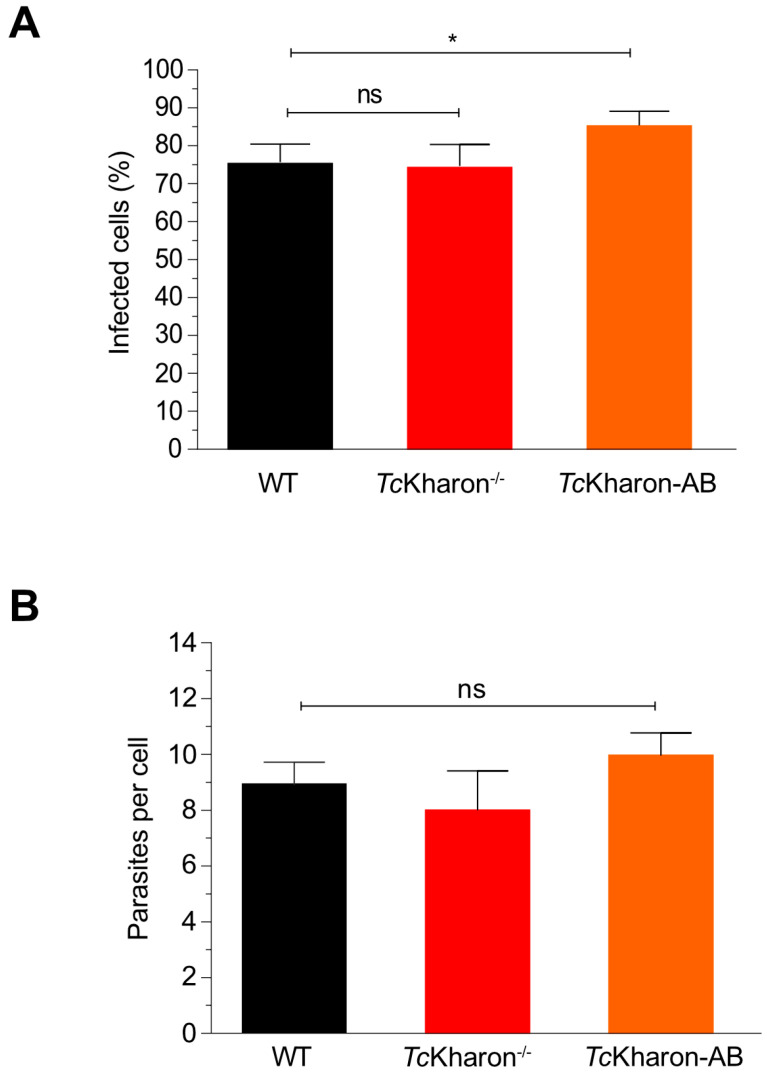
*Tc*Kharon disruption does not impair LLC-MK2 cell infection. (**A**) Tissue culture-derived trypomastigotes (TCTs) of *T. cruzi* WT, *Tc*Kharon^−/−^ and *Tc*Kharon-AB were incubated with LLC-MK2 cells for 5 h. (**B**) Quantification of amastigotes per cell. The asterisk represents a statistically significant difference at *p <* 0.05); ns represents a non-statistical difference.

Despite the morphological changes of *Tc*Kharon^−/−^ MTs this mutant was capable of infecting cell monolayers. Thus, the released tissue culture trypomastigotes (TCTs) could be used to perform infection assays. When LLC-MK2 cells were infected with WT, *Tc*Kharon^−/−^, or *Tc*Kharon-AB TCTs all three presented similar infection rates and numbers of amastigotes per host cell (Figure 8A,B). These data indicate that, despite the morphological alterations caused by *Tc*Kharon disruption, the parasites are able to achieve similar infection rates in vitro.

## 4. Discussion

Previously, through Western blot and immunofluorescence analysis, *Lmx*Kharon and *Tb*Kharon have been identified as being associated with the cytoskeleton, mainly in the subpellicular microtubules [17]. Here, we show that *Tc*Kharon is associated with the *T. cruzi* subpellicular microtubule cytoskeleton, like its *L. mexicana* and *T. brucei* orthologs. *Tb*Kharon is also associated with the spindle [17]. Due to the low level of mitotic cells in the unsynchronized *T. cruzi* epimastigote culture and strong subpellicular microtubule signal, we could not identify whether *Tc*Kharon is associated with the mitotic spindle.

Understanding Kharon deletion and knockdown phenotypes is complicated by Kharon’s multifunctional nature. It was originally discovered from its role in the correct localization of a protein (*Lm*xGT1) to the flagellar or pellicular plasma membrane in *L. mexicana* [16] and similarly in *T. brucei Tb*Kharon is necessary for the correct localization of the *Tb*CaCh protein to the flagellar membrane. However, not all flagellar membrane proteins require Kharon for correct localization, including FCaBP and SMP-1 [16,17]. Furthermore, RNAi knockdown in *T. brucei* caused flagellum detachment then disruption of mitotic spindles, although only at later time points [17].

The lethality of Kharon deletion or knockdown arises through cell division defects. In *L. mexicana*, the deletion of *Lmx*Kharon did not impair promastigote proliferation, but in amastigotes it led to an increased number of multinucleated cells through cell division failure, leading to a collapse in host macrophage infection [18]. Similar defects were observed in in vivo experiments [18]. RNAi knockdown of *Tb*Kharon in *T. brucei* led to a lethal phenotype, with the presence of multinucleated cells in both bloodstream and procyclic form trypomastigotes, suggesting a failure of cytokinesis [17]. Here, we show that *Tc*Kharon can be deleted and is not essential for *T. cruzi* epimastigotes, trypomastigotes or amastigotes in vitro. Nonetheless, it did cause a mild defect in growth rate and a tendency for cells with an abnormal nucleus and kinetoplast number.

In the cell cycle of *T. cruzi*, the kinetoplast (K) replicates before the nucleus, meaning that epimastigote cells containing one nucleus and one kinetoplast (1N1K) become 1N2K cells, and then 2N2K cells, followed by cytokinesis [40]. In this work, *Tc*Kharon^−/−^ epimastigote culture has an increase in 2N1K and 2N0K cells, which does not match the normal separation of organelles during the cell cycle [41]. This change may explain the mild growth defect of *Tc*Kharon^−/−^ culture. Furthermore, we observed that *Tc*Kharon^−/−^ cells are smaller compared to WT, and show a clear reduction in the distance from the nucleus to the posterior tip. The cells incorporate new tubulin units into old microtubules and regulate microtubule elongation at the posterior end to maintain cell shape and size, so the shrinkage of *Tc*Kharon^−/−^ cells likely indicates defective pellicular array growth [42,43].

Another noticeable feature of the *Tc*Kharon^−/−^ parasites is their larger and rounded posterior end, and the functional analysis of MAPs from *T. brucei* revealed similar features. The silencing of the WCB protein through RNAi caused this kind of phenotype since the cells became more rounded, with a slower growth rate, zoids (non-nucleated cells), and polynucleated cells, all of which show that cytokinesis fails in both procyclic and bloodstream forms [44]. Protein distribution and regulation play a crucial role in maintaining the shape and organization of subpellicular microtubules. In *T. brucei*, the overexpression of two MAPs (CAP15/CAP17) leads to aberrant cells that have an abnormal number of kinetoplasts and nuclei. This means that there is a problem with segregation coordination during cytokinesis, which causes mislocalization of organelles [45].

Why Kharon deletion causes cytokinesis defects is unclear. Given its prominent localization to the subpellicular array, the most straightforward conclusion is that it directly affects the stability of subpellicular microtubules or the regulation of morphogenesis [17]. However, the possibility of cytokinesis defects arising indirectly as a result of altered membrane composition has been proposed [17]. Our data support the idea that *Tc*Kharon plays a key, but not essential, role as an MAP in organizing the subpellicular array. However, we did not analyze whether flagellar membrane targeting of proteins is affected by Kharon deletion in *T. cruzi*, and thus cannot fully address this possibility.

*Tc*Kharon has no detectable orthologs outside of parasitic kinetoplastids, and our multiple sequence alignment analysis shows a low primary sequence conservation when compared to its orthologs in other kinetoplastids. However, Kharon has intriguing similarities to human Tau, a microtubule-binding protein highly expressed in neurons. Like Kharon, Tau is predicted to be almost entirely an IDP (intrinsically disordered protein) and binds to a parallel array/bundle of microtubules. Tau is very rich in proline and charged amino acids, similar to Kharon: 12.2 and 11.4% proline, 24.5 and 30.1% charged amino acids for Tau and *Tc*Kharon, respectively. This suggests similarity in IDP MAPs across extremely large evolutionary distances.

Kharon-deficient *L. infantum* has been demonstrated as a candidate for a live attenuated vaccine for leishmaniasis, since the gene deletion impairs amastigote replication [14]. Here, we were able to obtain trypomastigote *T. cruzi* from monolayers infected with MTs, providing strong but indirect evidence that *Tc*Kharon deletion is not lethal in intracellular amastigotes. It is possible that there are morphological changes to the amastigotes which would detrimentally affect in vivo infection. However, our data suggest *Tc*Kharon deletion is not a good route to generate a live attenuated *T. cruzi* vaccine.

Our analysis of the Kharon deletion phenotype in *T. cruzi* completes the picture of Kharon function in the different life cycle stages of all three human-infective trypanosomatid parasites. This revealed surprisingly large species to species and life stage to stage differences in how important Kharon is for cell division. This highlights the need to dissect the key players in cytoskeletal morphogenesis to explore possibilities for live attenuated vaccine development.

## Data Availability

No new data were created or analyzed in this study. Data sharing is not applicable to this article.

## Supplementary Materials

The following supporting information can be downloaded at: https://www.mdpi.com/article/10.3390/pathogens14040312/s1, Figure S1: Multiple alignment of *Tc*Kharon orthologs and identity matrix; Figure S2: Structure prediction and protein sequence alignment of Kharon orthologs; Figure S3: PONDR^®^ prediction (A–E), proline and charged amino acids compositions (F) of Kharon orthologs.; Figure S4: TEM images of *Tc*Kharon^−/−^ mutant cells with 2N1K configuration; Figure S5: Analysis of fluorescence of the *Tc*Kharon-AB cells [46,47].
