# Supplementary material for: Kharon Is Crucial for Trypanosoma cruzi Morphology but Does Not Impair In Vitro Infection
Source: Pathogens. 2025 Mar 25;14(4):312. doi: 10.3390/pathogens14040312 (PMC12030701; doi:10.3390/pathogens14040312)
Supplement: Supplementary file 1 [file pathogens-14-00312-s001.zip › pathogens-3499155-supplementary.pdf]

SUPPLEMENTARY FIGURES

pathogens

MDPI

Kharon is crucial for *Trypanosoma cruzi* morphology but does not impair *in vitro* infection

Jose Luis Saenz-Garcia [1&], Normanda Souza-Melo [2,3&], Juliana Severo Miranda [1], Beatriz Borges [4], Lisandro A. Pacheco-Lugo [5], José M. Quintero-Solano[6], Nilmar Moretti [3], Richard Wheeler [7], Lia C. Soares-Medeiros [4] and Wanderson D. DaRocha [1,*].

[1]Laboratório de Genômica Funcional de Parasitos (GFP), Universidade Federal de Paraná, Curitiba, Brasil.
[2]Laboratório de Ultraestrutura Hertha Mayer. Universidade Federal do Rio de Janeiro (UFRJ), Rio de Janeiro, Brasil.
[3]Laboratório de Biologia Molecular de Patógenos (LBMP) - Departamento de Microbiologia, Imunologia e Parasitologia – Escola Paulista de Medicina – Universidade Federal de São Paulo, São Paulo, Brasil.
[4]Laboratório de Biologia Celular, Instituto Carlos Chagas, Fundação Oswaldo Cruz (Fiocruz), Curitiba, Brasil.
[5]Facultad de Ciencias Básicas y Biomédicas. Universidad Simón Bolívar. Barranquilla, Colombia
[6]Laboratorio de Biotecnología Farmacéutica, Centro de Biotecnología Genómica, Instituto Politécnico Nacional, Reynosa, México.
[7]Nuffield Department of Medicine, University of Oxford, Oxford OX1 3SY, United Kingdom.
[&] These authors equally contributed to this work.
* Correspondence: Corresponding author: Wanderson D. DaRocha – wandersondarocha@gmail.com
Lia C. Soares-Medeiros – lia.medeiros@fiocruz.br

Supplementary figure S1A. ***Continue***

A

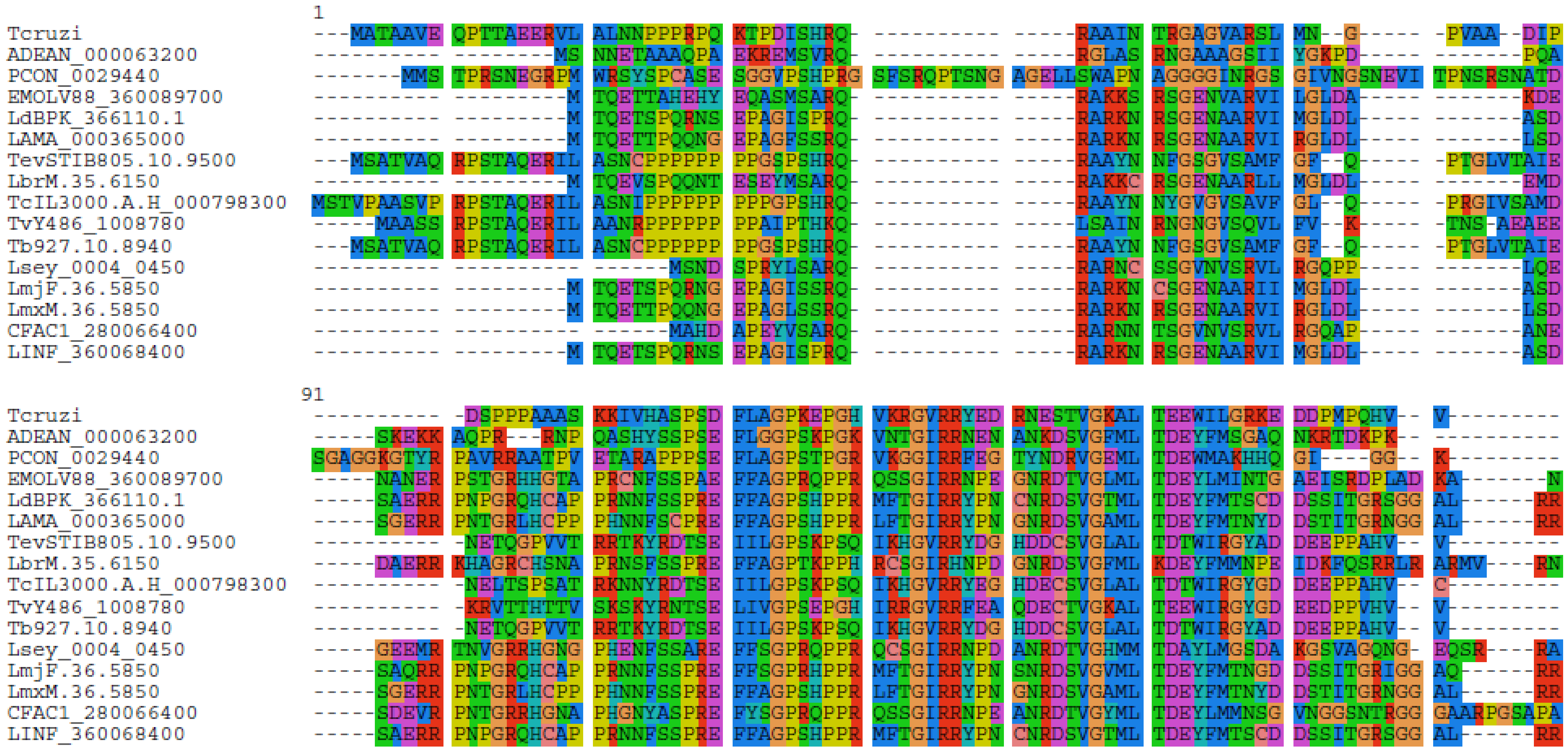

A

Supplementary figure S1A. *Continue*

```
                       181
Tcruzi                 ---------- ---------- ---------- ---------- -------RQR GPKDNLEGLC CSVRTEEAKP RLQRAPGLAP PHTTAAPYSE
ADEAN_000063200        ---------- ---------- ---------- ---------- -------VSR HNKSAM-THG LQVTEEPPPF RSGCKITHVQ GPMDCPYFED
PCON_0029440           ---------- ---------- ---------- ---------- -------RRY EQDPNLKGMA CEVVAEEAPM RQPVRLHPKY YSTVAPFYEG
EMOLV88_360089700      NKDSHDSHPQ MVASYGSPAK RGDGAIRQKK IRSQ--GKNK NQTIGATALF RHADGL-TNN CIVTAQEAPW RCGVKMTHPQ DPLGAPYFED
LdBPK_366110.1         -ASSRSLMYA ASGAYGTQAK RGEGTSRQKK ARKR--G--- GSINGATALL RCRDGL-TNN CIVTAPEAPW RCGVKITHPQ GAMEAPYFED
LAMA_000365000         -GSSRSPMRG GSGAYGSVAK RGEGALRQKK TRRR--A--- NSINGATAQF RCRDGL-TNN CIVTAPEAPW RCGVKITHPQ GGIDAPYFED
TevSTIB805.10.9500     ---------- ---------- ---------- ---------- -------RQR GPKDNLEGNC CRVKTEETKP RKMLAPGNPP PRTTRPPYSV
LbrM.35.6150           GSPCRYGSIS GMGAYGIQAR RGEGSLRQNK TLKM--ARM- NGSPWGTGSY HCRDGL-TNN CIVTAPEVPW RCGVRITHPQ GAMEAPYFED
TcIL3000.A.H_000798300 ---------- ---------- ---------- ---------- -------RRS GPMDNLEGNC CRVKTEEPKQ RKMLAPGNPP PRTTMPPYSV
TvY486_1008780         ---------- ---------- ---------- ---------- -------RQR GPKDNLEGNC CRILTEEAKP RKMRAPGNAP PHTSRAPYSE
Tb927.10.8940          ---------- ---------- ---------- ---------- -------RQR GPKDNLEGNC CRVKTEETKP RKMLAPGNPP PRTTRPPYSV
Lsey_0004_0450         AAARDEPASQ GAQPYGVAAK RGEGATRARN ELNAKRAAAY N--NTARAPN GSREGI-TYN CIVTAAEKEW RSGVKITHPQ GPMDVPYFED
LmjF.36.5850           -ASLHNLMYA SGDAYGAVAS RGQGASRQKK PRTR--G--- GSVNGATAPF RGRDGL-ANN CIVTAPEAPW RCGVKITHPQ GAMEAPYFED
LmxM.36.5850           -GSSRSPMRG GSGAYGSVAK RGEGALRQKK AHRR--A--- NSINGATAQF RCRDGL-TNN CIVTAPEAPW RCGVKITHPQ GGMDAPYFED
CFAC1_280066400        GGNANGQPAR GGQGYGAAAK RGQGTARARD AYNASRASNN QAVAAAHAPG GHREGL-TYN CIVTAPEKEW RSGVKITHPA GGMDAPYFED
LINF_360068400         -ASSRSLMYA ASGAYGTQAK RGEGTSRQKK ARKR--G--- GSINGATALL RCRDGL-TNN CIVTAPEAPW RCGVKITHPQ GAMEAPYFED

                       271
Tcruzi                 TELPPRPAEA VPVKGKRRFH --EQTSVNLF GRTAIST--R QAE--ADR-- -KAERLLHSR RNKANESLDV LNLGQYTRED LNEVE-RKIV
ADEAN_000063200        ND-PKPPRDE PAPTGKRHVK PRRNKDDQIF LEPGSP---- ------D--- -AKPLPKTNR TCKANKSVDV TNLSRYTAEE LKEAPERQTV
PCON_0029440           PDQTTSTRCN SSPQGRRRIA --TAGNGDFI SQYPQSTPRG PNGGEADSSS LSRPSTQPTR RYAQNRSLDV LNLGQYSPSH LGEKPIRTAM
EMOLV88_360089700      TD-PRPPRDV AAVTGKRRVK PPYKD-SKIM GQMPPLK--- --EGDEE-AL RAVLQPCHKS SNKTNESVDV LNLYTYTAEE LNEKPQPYKQ
LdBPK_366110.1         ND-PRPPRDA PPVTGKRHVP PPHKD-AKMF GQVPPPQ--- --EGEED-LF QSTLPPCHKT SNKANESVDV LNLHSYTADE LNEKPQPFKQ
LAMA_000365000         ND-PRPPRNV PPVTGKRHVP PPQKD-AKMF GQVPPPK--- --EGE-D-VF QSSLPPCHKT SNKANESVDV LNLHYYTADE LNEKPHPVKQ
TevSTIB805.10.9500     PEYDGNN--A EETCPKRLYR --HKDSGNII CPEPLPK--G TV---IDD-- -RPRGTVRSH RNRSNESVDV LNLGQYSSEQ LNEVE-RKLV
LbrM.35.6150           ND-PRPPRDV PAVTGKRHVK PPYKD-AKMF GQVPPPA--- --EGEED-EF HSILPPCHKT SNKANESVDV LNLYCYTADE LNEKPQPYKQ
TcIL3000.A.H_000798300 PEYGPVV--Q -EEAPKRRQV --RRDNGNII CPEPLPK--G TV---FDD-- -RKRGTVRTS RNRSNESVDV LNLGQYTREQ LNEVE-RKYI
TvY486_1008780         PDLNDLYVSS EQNVPRRQYV --CRDNGNII CPDPLPH--G TV---LDD-- -RPRGTVRSH CNRSNMSVDV LNLGQYTREE LNEVE-RKIV
Tb927.10.8940          PEYDGNN--A EETCPKRLYR --HKDSGNII CPEPLPK--G TV---IDD-- -RPRGTVRSH RNRSNESVDV LNLGQYSSEQ LNEVE-RKLV
Lsey_0004_0450         ND-PRPARDV PTATGKRRVR PPYKD-AKMF GQMALLP--- --EGEEGKDN EKVLQPCHKK ANKANESVDV LNLHCYSAEE LSEKPQPHKQ
LmjF.36.5850           ND-PRPPRDA PPTTGKRHVP PPHKD-AKMF GQAPPLR--- --EGE-D-VL QSSLPPCHKA SNKTNESVDV LNLHCYTADE LNEKPPSFKQ
LmxM.36.5850           ND-PRPPRNV PPVTGKRHVP PPQKD-AKMF GQVPPPK--- --EGE-D-VF QSSLPPCHKT SNKANESVDV LNLHYYTADE LNEKPHPVKQ
CFAC1_280066400        TD-PRPARDA PAVTGKRHVR PPQKD-AKMF GQVPPPK--- --EGEEAEAI QSCLPPCRKS SNKANESVDV LNLYCYSPEE LSEKPQPYKQ
LINF_360068400         ND-PRPPRDA PPVTGKRHVP PPHKD-AKMF GQVPPPQ--- --EGEED-LF QSTLPPCHKT SNKANESVDV LNLHSYTADE LNEKPQPFKQ

                       361
Tcruzi                 LGPRQFIPAS LPPRQRPIIA RLKSHANETH DIFGTG-KGV EDVEPVHRKN PGASAPKNSC AANIFGGPPL EQKPPRPFRP SQLLTYEEAP
ADEAN_000063200        LGPRKFVAPD MPPAKPPAIK PINTAAKQDH DVLGDGRFGK PEEAKPAGKG QGSCRPPKQQ ANLFAGPPPK KKGGRGSLA- ----------
PCON_0029440           TGPPIFIPAP LPPRKPSSLR RTNSPARVDH DIFGTG-WGR SEVVHPHSRG RGDCCPEPVV TPAFLEIPKP ATISTSESAP SPMPKR----
EMOLV88_360089700      LGPRVCPPPD MPPKKPAVIK PINTPAKQEH DVLGTGRWGV PAKEQPHGLA RGACRPPRDT ANLFRGGIPQ NHHKGSSPD- ----------
LdBPK_366110.1         LGPRKCSAPE LPPRKPPVIK PINTPAKQEH DVLGTGRWGS PEKEQPRGLA RGLCRPPHDT ANLFHGGMPQ PDEKAGSTRP QESVGSKGGS
LAMA_000365000         LGPRKCESQE LPPRKPPVIK PINTPAKQEH DVLGTGRWGF PEKEHPRGLA RGLCRPPHDT ANLFYGGMLL ADDNAGSARH QESVGNKRGS
TevSTIB805.10.9500     AGPREFIPPP LPPRQRPIIA RVHTPANATH DIFGTG-QGV EDIEPVHCKK RGESAPKRSG EFDIFDPTPL PQQPTKGYNP SDLLVYDEPY
LbrM.35.6150           LGPRKCGAPE LPPKKPPVIK HIDTPARQEH DVLGTGRWGF PEKQVPRGLA RGLCRPRHDT ANLFYGGPAQ EDDKLASGTP RESAGRNGGS
TcIL3000.A.H_000798300 VGPREFIPES LPPRQRPIIA RVHTPANATH DIFGTG-NGV EDVEPVHCKK RGESAPKRSN EFDIFDPTPA PQNPPKPYCP SDLLVYDEPR
TvY486_1008780         AGPRAFTPPP LPPRQRALIS RVRTNANVTH DIFGTG-NGV DDAEPIHCKR NGECAPKRAD EFSIFDTTPL PQKPVKEFYP SDLLAYNEPR
Tb927.10.8940          AGPREFIPPP LPPRQRPIIA RVHTPANATH DIFGTG-QGV EDIEPVHCKK RGESAPKRSG EFDIFDPTPL PQQPTKGYNP SDLLVYDEPY
Lsey_0004_0450         LGPRKYVAPD MPPRKPPMIH PINTVAKQEH DVLGTGRWGV PEKEHPHGLA RGSCRPPHDT ANLFGGDKR- ---------- ----------
LmjF.36.5850           LGPRKCSAQE LPPRKPPVIK PINTPAKQEH DVLGTGRWGL PEKEQPRGLA RGLCRPPHDT ANLFYGGMLP PDEKADSACL QEPVGSKGGR
LmxM.36.5850           LGPRKCESQE LPPRKPPVIK PINTPAKQEH DVLGTGRWGF PEKEHPRGLA RGLCRPPHDT ANLFYGGMLL ADDNAGSARH QESVGSKPGS
CFAC1_280066400        LGPRKYVAPD MPPRRQPMIH PINTPAKQEH DVLGTGRWGI PEKEHPRGLA RGACRPPRDT ANLFRGGIAP AEESAPRSY- ----------
LINF_360068400         LGPRKCSAPE LPPRKPPVIK PINTPAKQEH DVLGTGRWGS PEKEQPRGLA RGLCRPPHDT ANLFHGGMPQ PDEKAGSTCP QESVGSKGGS
```

Supplementary figure S1A.

A

```
                       451
Tcruzi                 NLSKKRKDLV -EAPA----- --RPA-SANA AASRSGKKLE AGQLFNSKSP ----DIE-PR G--------- GRSRG----V YAPKT-----
ADEAN_000063200        ---------- ---------- ---------- ---------- ---------- ---------- ---------- ---------- ----------
PCON_0029440           ---------- ---------- ---------E SSFHIGG--- -----DDRSS ----TQV-QR R--------- TRASG----V YSPGR---YK
EMOLV88_360089700      ---------- ---------- ---------- ---------R TGNAPGNRSA SQQSRKEDLH HNDGHSASSE KRGDAALDDK NRVRKQLSKK
LdBPK_366110.1         LSARTDSRQP RVIDSRRTGS RSGSVSARTD QVYAIQGEPH RASSPGSRVA ARPSRQVEAY HVDSRSRSPK KREDPVFDDS YRPHRYLFKK
LAMA_000365000         LSARTDQRHP RAMDSRRTRS PSGSVSARTD SRYPAQG--- ------SRVT AHLSRQGEAH HGSSRSSSPK KRADPVFDDN YRPHRMVFKK
TevSTIB805.10.9500     VSVQEKREKA -AREARRNPE KYCPG-NVVT SSHKIEKKTE SGVVFHSKSQ ----EIT-AH G--------- GRARG----L YAPRG-----
LbrM.35.6150           NGAHLSPRQS HQGESY---- ---------- -----RSASR RSSSHGSRTS ARPSRQVQPF RA-HRNTPPK KREDPVFDSN FRPHKVVFK-
TcIL3000.A.H_000798300 VSSKELRNQA -AREARRNPE KYCPV-TTVM SPQKIAKKTE NGVMFHSKSQ ----EIT-PR G--------- GRARG----I YAPKP-----
TvY486_1008780         VSAKELREKA -AREARMKPV P-CTA-FQTP GSNKIEGRVE RGLLYTPKSS ----EIV-AR G--------- GRARG----T YAPKA-----
Tb927.10.8940          VSVQEKREKA -AREARRNPE KYCPG-NVVT SSHKIEKKTE SGVVFHSKSQ ----EIT-AH G--------- GRARG----L YAPRG-----
Lsey_0004_0450         ---------- ---------- ---------- ---------- ---------- ---------- ---------K RRALPVPTDS NHPSKVLSKK
LmjF.36.5850           VSARTDRRHP ---------- ---------- ----IQGEPR S--------- ---------- -----VSSPK KRDDPVFDDN YRPHKHMFKK
LmxM.36.5850           LSARTDQRHP RAMDSRRTRS PSGSVSARTD SRYPAQG--- ------SRVA AHPSRQGEAH HGSSRSSSPK KRADPVFDDN YRPHRMVFKK
CFAC1_280066400        ---------- ---------- ---------- -----QPAPR RGS-VEAPSE RRSSRQVEPF RVDLRGGSPK KRPDPVFTDA DRPRKILSKR
LINF_360068400         LSARTDSRQP RVIDSRRTGS RSGSVSARTD QVYAIQGEPH RASSPGSRVA ARPSRQVEAY HVDSRSRSPK KREDPVFDDS YRPHRYLFKK

                       541
Tcruzi                 --GINNIF-- ---------- ---------- ---------- ---------- ---------- ---------
ADEAN_000063200        PESKGNILKY YDPATDNAAD FAPPPSRHRE NKSNKESKPE ---PEKPVHK N----TKSNV SSQKNLW--
PCON_0029440           NASSINIFSW N--------- ---------- ---------- ---------- ---------- ---------
EMOLV88_360089700      NAGSTNILRY YDPSVDPVPA K-PQVR--GT ARANVESKLD DSNTPYVIGK ARGEFYRNSV SSITLV---
LdBPK_366110.1         NAGNPNILHY YDPTIDPMPA A-APKR--PI PRSNMESKLD DLNTPQVIGK ARGEFYKNSR SSIALI---
LAMA_000365000         NAGDPNILHY YDPNIDSMPA A-PPRR--PI PRSNMESHLD DLNTPHTIGR ARGEFCKNSR STIALV---
TevSTIB805.10.9500     --SSISLC-- ---------- ---------- ---------- ---------- ---------- ---------
LbrM.35.6150           NVGNPNLLRY YDPTVDPMPE AMAPKR--SA PRSNIESMLD DSNMPCAFGK GRGEFNKYSR STITLV---
TcIL3000.A.H_000798300 --TAISLC-- ---------- ---------- ---------- ---------- ---------- ---------
TvY486_1008780         --CALSLY-- ---------- ---------- ---------- ---------- ---------- ---------
Tb927.10.8940          --SSISLC-- ---------- ---------- ---------- ---------- ---------- ---------
Lsey_0004_0450         NAGSPELLRY YDPNVDPKPV PNATKR--NG QRCNLETREL LPSERRPTGK GRGEFAKNSH DSVVLAYA-
LmjF.36.5850           NAGSPNILRY YDPTIDPTPA A-SPKR--PI PRSNMESKLD DLNAPQVIGK GRGEFYKNSR SSIALM---
LmxM.36.5850           NAGDPNILHY YDPNIDSMPA A-PPRR--PI PRSNMESHLD DLNTPHTIGR ARGEFCKNSR STIALV---
CFAC1_280066400        NAGSPDLLRY YDPNVDPKPA QSPPNR--NG QRANQETREL TAAEKRPSGK GRGEFASNNR DTVSLTYSS
LINF_360068400         NAGNPNILHY YDPTIDPMPA A-APKR--PI PRSNMESKLD DLNTPQVIGK ARGEFYKNSR SSIALI---
```

Supplementary figure S1B

B

GENE ID		*T. cruzi*	*T. congolense*	*T. vivax*	*T. evansi*	*T. b. brucei*	*P. confusum*	*E. monterogeii*	*L. major*	*L. donovani*	*L. infantum*	*C. fasciculata*	*L. seymouri*	*L. mexicana*	*L. amazonensis*	*A. deanei*	*L. braziliensis*
C4B63_14g70	*T. cruzi*	100.00															
TcIL3000.A.H_000798300	*T. congolense*	47.38	100.00														
TvY486_1008780	*T. vivax*	45.64	60.59	100.00													
TevSTIB805.10.9500	*T. evansi*	45.27	78.05	61.43	100.00												
Tb927.10.8940	*T. b. brucei*	45.27	78.05	61.43	100.00	100.00											
PCON_0029440	*P. confusum*	30.14	29.48	29.32	29.12	29.12	100.00										
EMOLV88_360089700	*E. monterogeii*	27.60	24.92	23.51	25.75	25.75	27.01	100.00									
LmjF.36.5850	*L. major*	27.38	27.41	23.12	26.74	26.74	29.48	60.35	100.00								
LdBPK_366110.1	*L. donovani*	26.98	25.98	22.98	25.13	25.13	29.48	60.13	84.82	100.00							
LINF_360068400	*L. infantum*	26.72	26.25	22.98	25.13	25.13	29.48	60.13	85.03	99.81	100.00						
CFAC1_280066400	*C. fasciculata*	26.65	26.06	23.72	26.89	26.89	28.20	54.11	56.86	56.30	56.30	100.00					
Lsey_0004_0450	*L. seymouri*	26.64	25.67	22.77	26.25	26.25	26.17	54.27	55.94	56.51	56.51	67.65	100.00				
LmxM.36.5850	*L. mexicana*	26.36	25.88	22.79	25.27	25.27	29.92	59.02	79.25	82.50	82.31	56.75	56.64	100.00			
LAMA_000365000	*L. amazonensis*	26.09	25.88	22.79	25.27	25.27	29.92	58.81	78.83	81.54	81.35	56.10	55.94	98.27	100.00		
ADEAN_000063200	*A. deanei*	25.99	22.92	22.04	23.18	23.18	25.48	40.66	42.03	44.23	44.23	39.00	40.69	42.86	42.31	100.00	
LbrM.35.6150	*L. braziliensis*	25.68	22.31	19.95	21.15	21.15	27.25	55.00	63.26	66.60	66.60	53.54	53.56	64.73	64.33	41.64	100.00

Supplementary Figure 1. Multiple alignment of *Tc*Kharon orthologs and identity matrix. Panel **A** shows the multiple alignment of *Tc*Kharon orthologs using Seaview® software, and in **B** it is shown the identity matrix. This alignment was used to build the phylogenetic tree presented in Fig. 1A.

Supplementary figure S2A

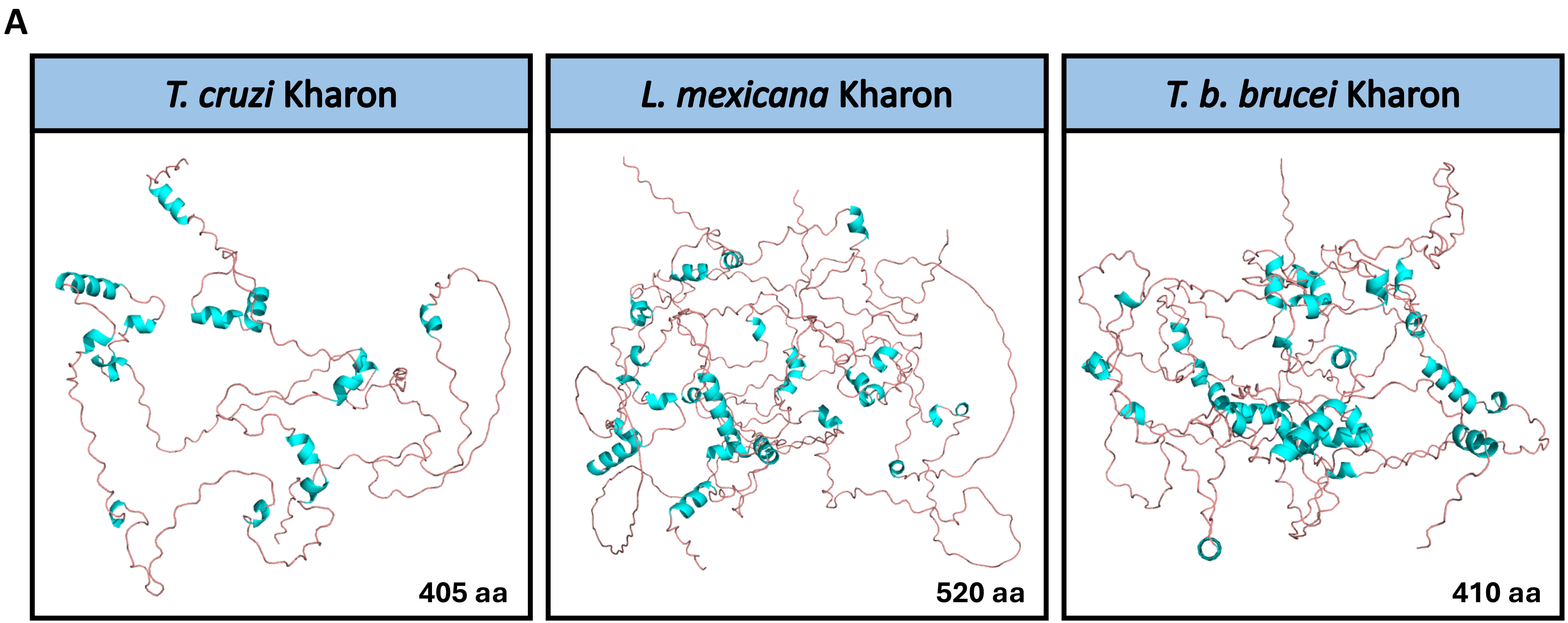

Supplementary figure S2B

B

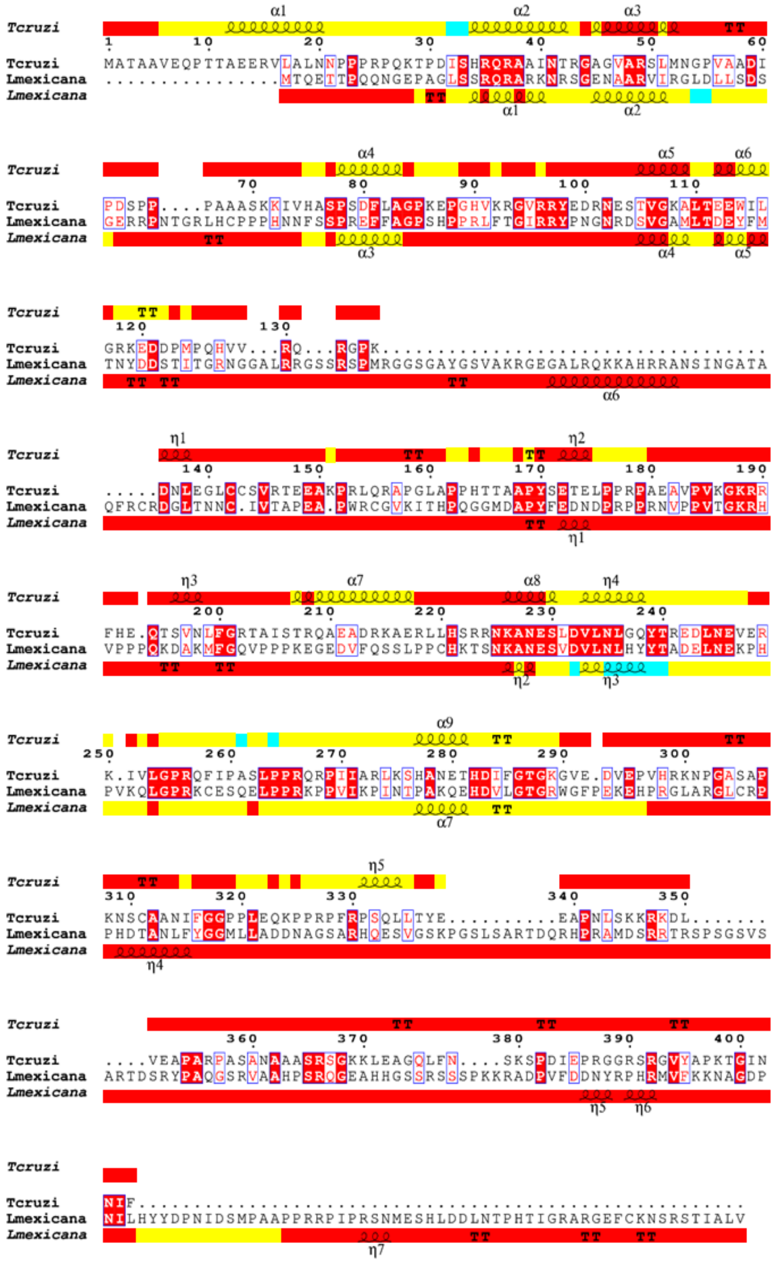

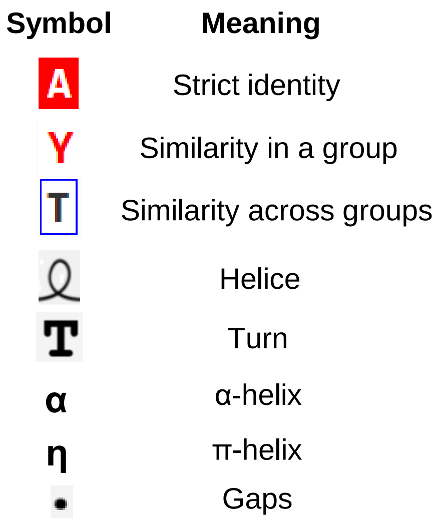

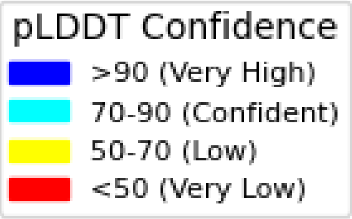

Supplementary figure S2C

C

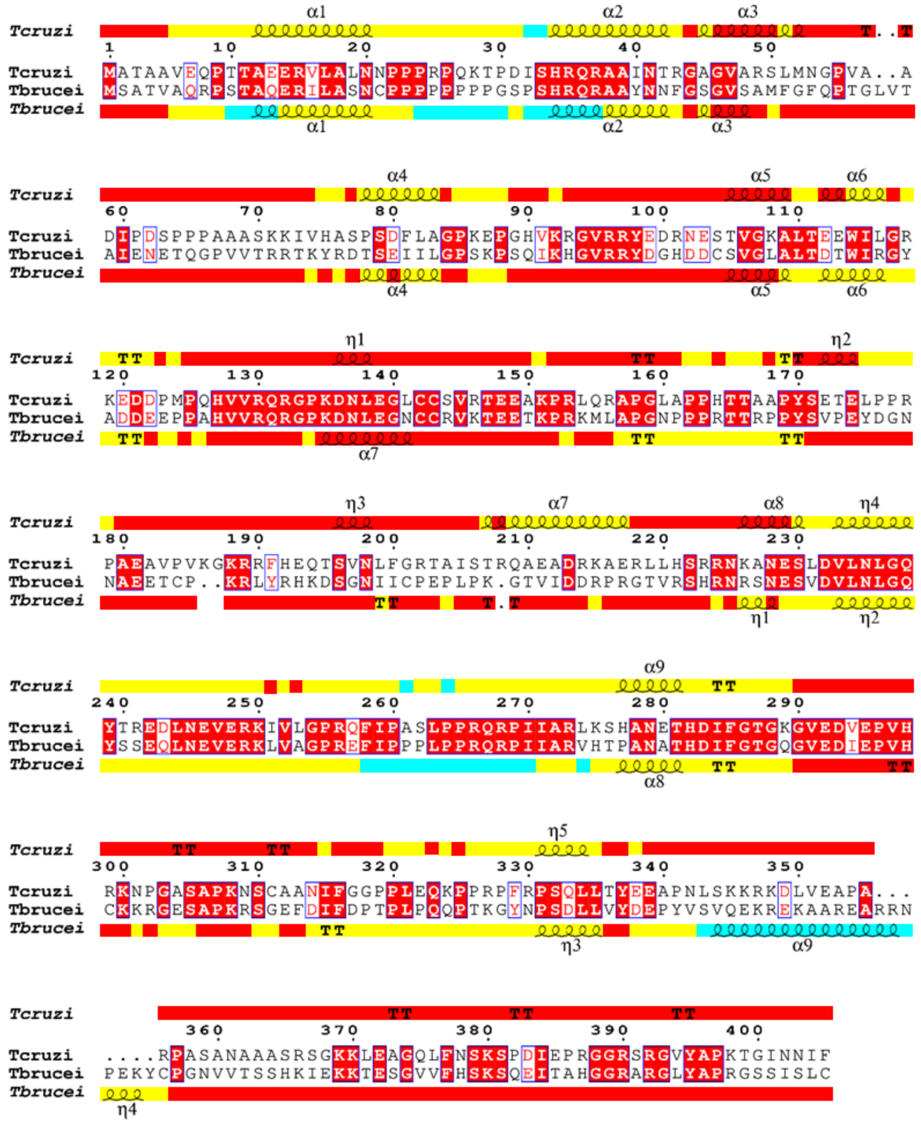

Supplementary Figure 2. Structure prediction and protein sequence alignment of Kharon orthologs. A. Structure prediction using AlphaFold3 [46] of *T. cruzi*, *L. mexicana*, and *T. b. brucei* Kharon proteins. The models were assigned with ranking scores of 0.61, 0.61, and 0.63, respectively. Cyan represents α-helix, and pink unfolded/loop regions. **B and C.** Protein sequences alignments of *T. cruzi* and *L. mexicana* (**B**); and *T. cruzi* and *T. b. brucei* (**C**) using Clustal Omega, and the alignments were visualized and analyzed with structural annotations using ESPript 3.0 (https://espript.ibcp.fr/ESPript/cgi-bin/ESPript.cgi) [47]. The color scheme and other features are detailed on the legend on panel **B**.

46. Abramson, J.; Adler, J.; Dunger, J.; et al. Accurate structure prediction of biomolecular interactions with AlphaFold 3. *Nature*. **2024**, 630(8016):493-500. doi: 10.1038/s41586-024-07487-w.

47. Robert, X; and Gouet, P. Deciphering key features in protein structures with the new ENDscript server. *Nucleic Acids Res*. **2014**, 42(Web Server issue):W320-4. doi: 10.1093/nar/gku316.

Supplementary figure S3A

A Kharon from *T. cruzi*

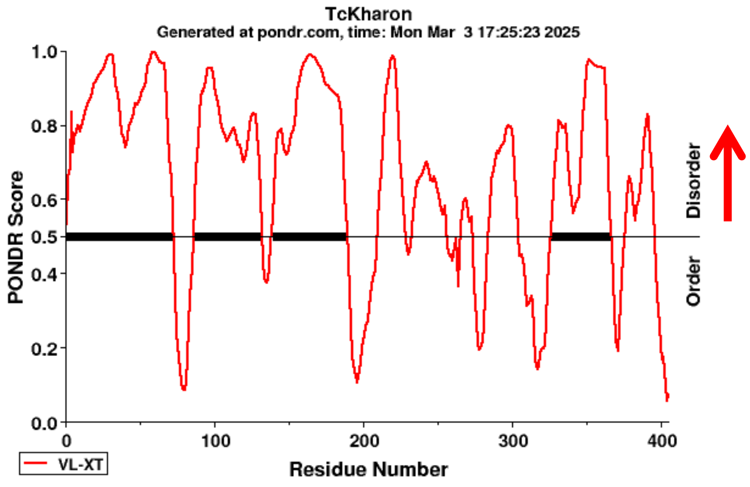


```
================================VLXT NNP STATISTICS========================
Predicted residues: 405               Number Disordered Regions: 9
Number residues disordered: 301       Longest Disordered Region: 72
Overall percent disordered: 74.32     Average Prediction Score: 0.6613
Predicted disorder segment [1]-[72]     Average Strength= 0.8639
Predicted disorder segment [87]-[132]   Average Strength= 0.7952
Predicted disorder segment [139]-[189]  Average Strength= 0.8458
Predicted disorder segment [210]-[228]  Average Strength= 0.7898
Predicted disorder segment [233]-[256]  Average Strength= 0.6259
Predicted disorder segment [266]-[273]  Average Strength= 0.5678
Predicted disorder segment [285]-[304]  Average Strength= 0.7094
Predicted disorder segment [326]-[366]  Average Strength= 0.7816
Predicted disorder segment [376]-[395]  Average Strength= 0.6559
```

Kharon from *C. fasciculata*

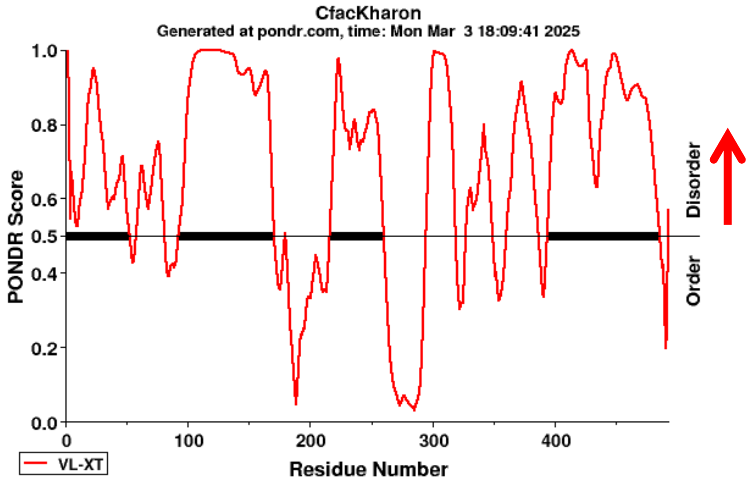


```
================================VLXT NNP STATISTICS================================
Predicted residues: 493               Number Disordered Regions: 10
Number residues disordered: 364       Longest Disordered Region: 93
Overall percent disordered: 73.83     Average Prediction Score: 0.6719
Predicted disorder segment [1]-[52]     Average Strength= 0.7040
Predicted disorder segment [58]-[81]    Average Strength= 0.6372
Predicted disorder segment [93]-[170]   Average Strength= 0.9175
Predicted disorder segment [180]-[180]  Average Strength= 0.5078
Predicted disorder segment [217]-[259]  Average Strength= 0.7820
Predicted disorder segment [295]-[318]  Average Strength= 0.8828
Predicted disorder segment [328]-[349]  Average Strength= 0.6403
Predicted disorder segment [361]-[386]  Average Strength= 0.7419
Predicted disorder segment [394]-[486]  Average Strength= 0.8547
Predicted disorder segment [493]-[493]  Average Strength= 0.5692
```

Supplementary figure S3B

B Kharon from *A. deanei*

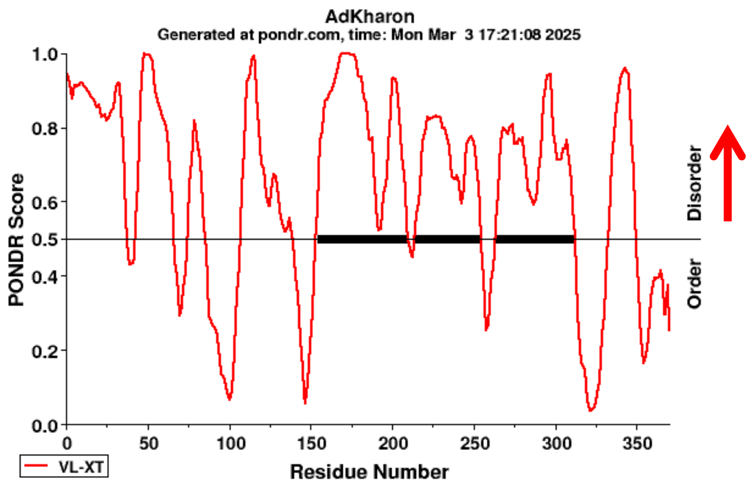


```
================================VLXT NNP STATISTICS================================
Predicted residues: 370                  Number Disordered Regions: 8
Number residues disordered: 267          Longest Disordered Region: 56
Overall percent disordered: 72.16        Average Prediction Score: 0.6403
Predicted disorder segment [1]-[37]      Average Strength= 0.8619
Predicted disorder segment [43]-[66]     Average Strength= 0.8315
Predicted disorder segment [75]-[85]     Average Strength= 0.6830
Predicted disorder segment [107]-[138]   Average Strength= 0.6979
Predicted disorder segment [154]-[209]   Average Strength= 0.8260
Predicted disorder segment [214]-[254]   Average Strength= 0.7258
Predicted disorder segment [264]-[312]   Average Strength= 0.7339
Predicted disorder segment [333]-[349]   Average Strength= 0.8120
```

Kharon from *L. seymouri*

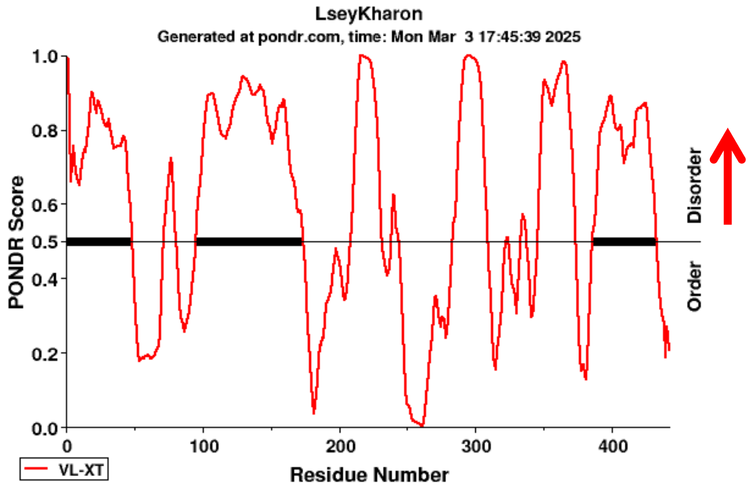


```
================================VLXT NNP STATISTICS================================
Predicted residues: 442                  Number Disordered Regions: 10
Number residues disordered: 271          Longest Disordered Region: 79
Overall percent disordered: 61.31        Average Prediction Score: 0.5972
Predicted disorder segment [1]-[48]      Average Strength= 0.7753
Predicted disorder segment [72]-[80]     Average Strength= 0.6314
Predicted disorder segment [96]-[174]    Average Strength= 0.8116
Predicted disorder segment [209]-[231]   Average Strength= 0.8548
Predicted disorder segment [239]-[243]   Average Strength= 0.5709
Predicted disorder segment [284]-[309]   Average Strength= 0.8533
Predicted disorder segment [323]-[324]   Average Strength= 0.5083
Predicted disorder segment [334]-[337]   Average Strength= 0.5419
Predicted disorder segment [346]-[373]   Average Strength= 0.8603
Predicted disorder segment [386]-[432]   Average Strength= 0.7755
```

Supplementary figure S3C

C Kharon from *T. b. brucei*

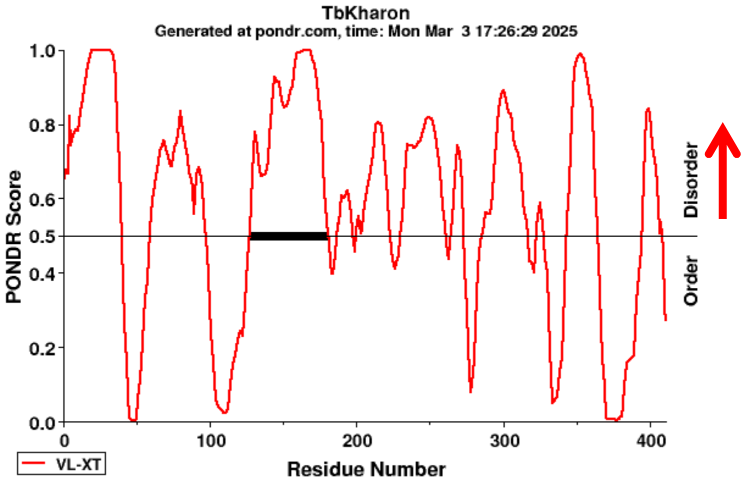


```
================================VLXT NNP STATISTICS========================
Predicted residues: 411                  Number Disordered Regions: 11
Number residues disordered: 277          Longest Disordered Region: 54
Overall percent disordered: 67.40        Average Prediction Score: 0.5831
Predicted disorder segment [1]-[39]      Average Strength= 0.8862
Predicted disorder segment [59]-[96]     Average Strength= 0.6885
Predicted disorder segment [127]-[180]   Average Strength= 0.8330
Predicted disorder segment [187]-[197]   Average Strength= 0.5772
Predicted disorder segment [200]-[223]   Average Strength= 0.6516
Predicted disorder segment [231]-[260]   Average Strength= 0.7267
Predicted disorder segment [265]-[273]   Average Strength= 0.6432
Predicted disorder segment [285]-[316]   Average Strength= 0.6992
Predicted disorder segment [323]-[327]   Average Strength= 0.5486
Predicted disorder segment [344]-[364]   Average Strength= 0.8426
Predicted disorder segment [395]-[408]   Average Strength= 0.6875
```

Kharon from *T. vivax*

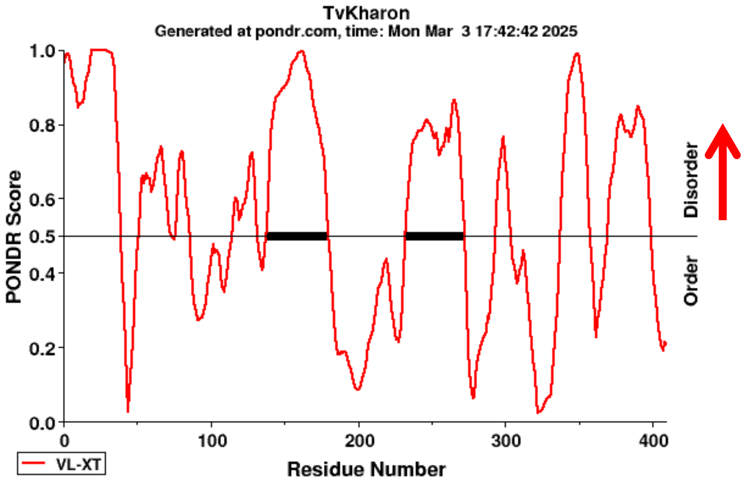


```
================================VLXT NNP STATISTICS================================
Predicted residues: 409                  Number Disordered Regions: 9
Number residues disordered: 232          Longest Disordered Region: 43
Overall percent disordered: 56.72        Average Prediction Score: 0.5624
Predicted disorder segment [1]-[39]      Average Strength= 0.9247
Predicted disorder segment [51]-[73]     Average Strength= 0.6364
Predicted disorder segment [76]-[85]     Average Strength= 0.6290
Predicted disorder segment [115]-[131]   Average Strength= 0.6072
Predicted disorder segment [138]-[180]   Average Strength= 0.8492
Predicted disorder segment [233]-[272]   Average Strength= 0.7533
Predicted disorder segment [294]-[304]   Average Strength= 0.6474
Predicted disorder segment [338]-[357]   Average Strength= 0.8384
Predicted disorder segment [370]-[398]   Average Strength= 0.7550
```

Supplementary figure S3D

D Kharon from *L. braziliensis*

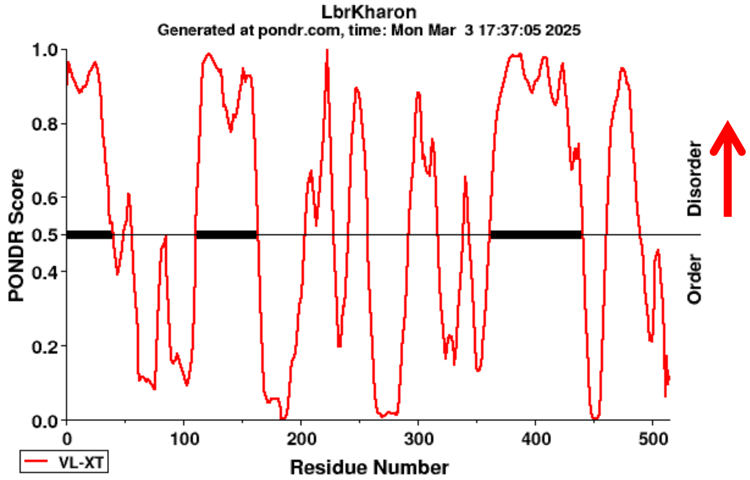


```
================================VLXT NNP STATISTICS================================
Predicted residues: 515                   Number Disordered Regions: 9
Number residues disordered: 278           Longest Disordered Region: 80
Overall percent disordered: 53.98         Average Prediction Score: 0.5350
Predicted disorder segment [1]-[40]       Average Strength= 0.8447
Predicted disorder segment [50]-[55]      Average Strength= 0.5585
Predicted disorder segment [111]-[164]    Average Strength= 0.8600
Predicted disorder segment [204]-[228]    Average Strength= 0.6774
Predicted disorder segment [242]-[257]    Average Strength= 0.7642
Predicted disorder segment [293]-[317]    Average Strength= 0.7097
Predicted disorder segment [340]-[343]    Average Strength= 0.6039
Predicted disorder segment [362]-[441]    Average Strength= 0.8646
Predicted disorder segment [462]-[489]    Average Strength= 0.8007
```

Kharon from *L. mexicana*

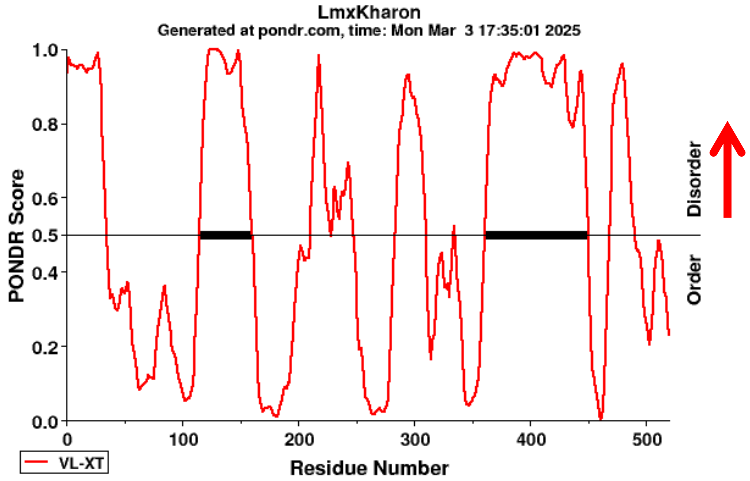


```
================================VLXT NNP STATISTICS================================
Predicted residues: 520                   Number Disordered Regions: 8
Number residues disordered: 253           Longest Disordered Region: 89
Overall percent disordered: 48.65         Average Prediction Score: 0.5288
Predicted disorder segment [1]-[35]       Average Strength= 0.9093
Predicted disorder segment [116]-[159]    Average Strength= 0.9066
Predicted disorder segment [211]-[227]    Average Strength= 0.7245
Predicted disorder segment [229]-[247]    Average Strength= 0.5984
Predicted disorder segment [284]-[309]    Average Strength= 0.7972
Predicted disorder segment [335]-[335]    Average Strength= 0.5220
Predicted disorder segment [362]-[450]    Average Strength= 0.9081
Predicted disorder segment [469]-[490]    Average Strength= 0.7836
```

Supplementary figure S3E

E Kharon from *L. amazonensis*

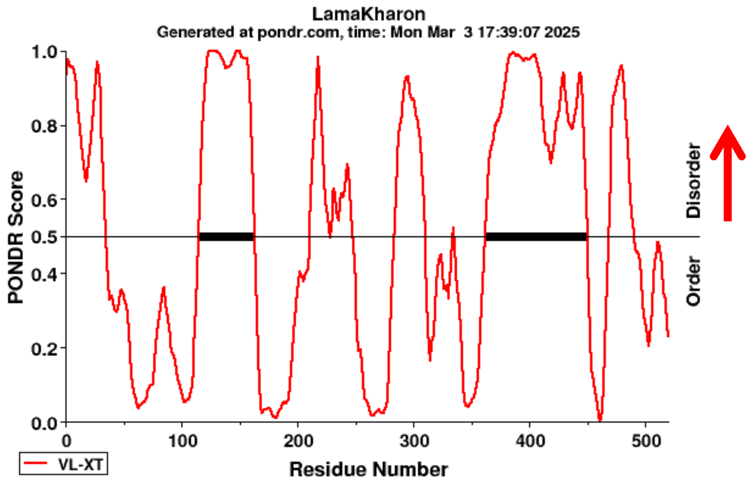


```
================================VLXT NNP STATISTICS================================
Predicted residues: 520                  Number Disordered Regions: 8
Number residues disordered: 257          Longest Disordered Region: 89
Overall percent disordered: 49.42        Average Prediction Score: 0.5152
Predicted disorder segment [1]-[35]      Average Strength= 0.8190
Predicted disorder segment [116]-[163]   Average Strength= 0.9295
Predicted disorder segment [211]-[227]   Average Strength= 0.7234
Predicted disorder segment [229]-[247]   Average Strength= 0.5984
Predicted disorder segment [284]-[309]   Average Strength= 0.7972
Predicted disorder segment [335]-[335]   Average Strength= 0.5220
Predicted disorder segment [362]-[450]   Average Strength= 0.8552
Predicted disorder segment [469]-[490]   Average Strength= 0.7836
```

Kharon from *L. infantum*

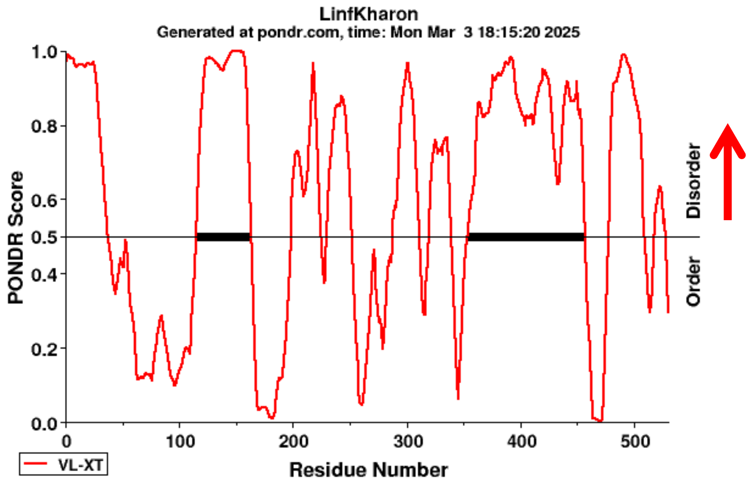


```
================================VLXT NNP STATISTICS================================
Predicted residues: 530                  Number Disordered Regions: 9
Number residues disordered: 321          Longest Disordered Region: 103
Overall percent disordered: 60.57        Average Prediction Score: 0.5943
Predicted disorder segment [1]-[37]      Average Strength= 0.8896
Predicted disorder segment [116]-[163]   Average Strength= 0.9276
Predicted disorder segment [199]-[224]   Average Strength= 0.7126
Predicted disorder segment [230]-[251]   Average Strength= 0.7629
Predicted disorder segment [288]-[310]   Average Strength= 0.7816
Predicted disorder segment [320]-[339]   Average Strength= 0.6998
Predicted disorder segment [354]-[456]   Average Strength= 0.8403
Predicted disorder segment [478]-[509]   Average Strength= 0.8629
Predicted disorder segment [518]-[527]   Average Strength= 0.5914
```

F

Species	Gene ID	Protein lenght (amino acids)	# of proline residues	% of proline	% of charged amino acids
Angomonas deanei	ADEAN_000063200	370	47	12.70	31.35
Crithidia fasciculata	CFAC1_280066400	493	57	11.56	28.19
Endotrypanum monterogeii	EMOLV88_360089700	483	45	9.32	30.23
Leishmania amazonensis	LAMA_000365000	520	56	10.77	29.62
Leishmania braziliensis	LbrM.35.6150	515	53	10.29	30.10
Leishmania donovani	LdBPK_366110.1	530	61	11.51	28.87
Leishmania infantum	LINF_360068400	530	61	11.51	28.68
Leishmania major	LmjF.36.5850	481	58	12.06	28.48
Leishmania mexicana	LmxM.36.5850	520	58	11.15	29.42
Leptomonas seymouri	Lsey_0004_0450	442	44	9.95	31.00
Paratrypanosoma. confusum	PCON_0029440	417	47	11.27	23.02
Trypanosoma brucei brucei	Tb927.10.8940	411	49	11.92	29.20
Trypanosoma congolense	TcIL3000.A.H_000798300	413	55	13.32	28.09
Trypanosoma cruzi	C4B63_14g70	405	46	11.36	30.12
Trypanosoma evansi	TevSTIB805.10.9500	411	49	11.92	29.20
Trypanosoma vivax	TvY486_1008780	409	44	10.76	29.34

Supplementary Figure S3. PONDR® prediction (A-E), proline and charged amino acids compositions (F) of Kharon orthologs. PONDR® (Predictor of Natural Disordered Regions - www.pondr.com) was used to predict disordered and ordered regions in Kharon proteins from *T. cruzi*, and *C. fasciculata* (**3A**); *A. deanei*, and *L. seymouri* (**3B**); *T. b. brucei*, *T. vivax* (**3C**); *L. braziliensis*, and *L. mexicana* (**3D**); *L. amazonensis*, and *L. infantum* (**3E**). It is shown side by side, for each ortholog, graphical and textual representation.

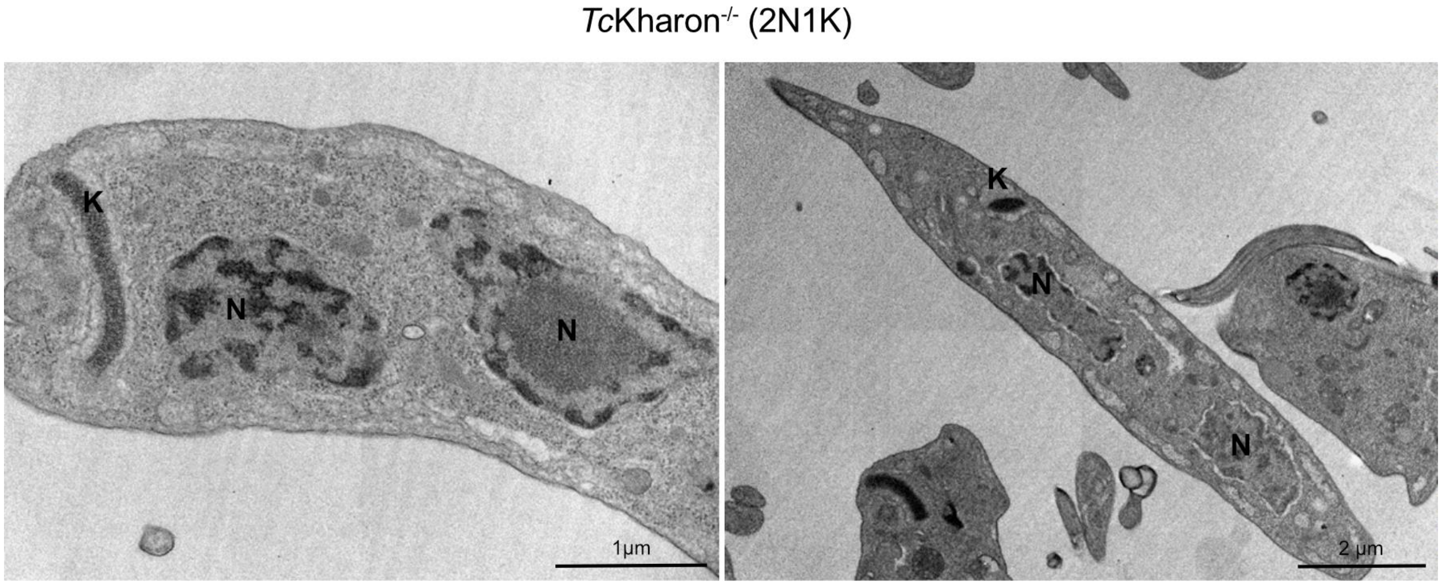

Supplementary Figure S4. TEM images of *Tc*Kharon$^{-/-}$ mutant cells with 2N1K configuration.

Supplementary figure S5

A

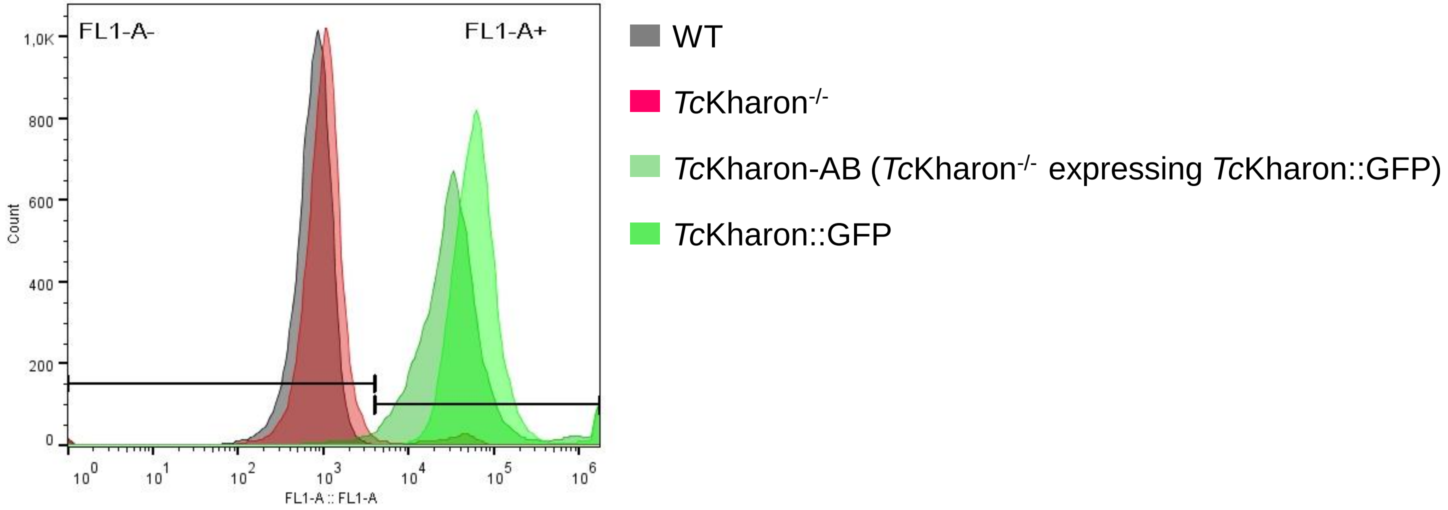

B

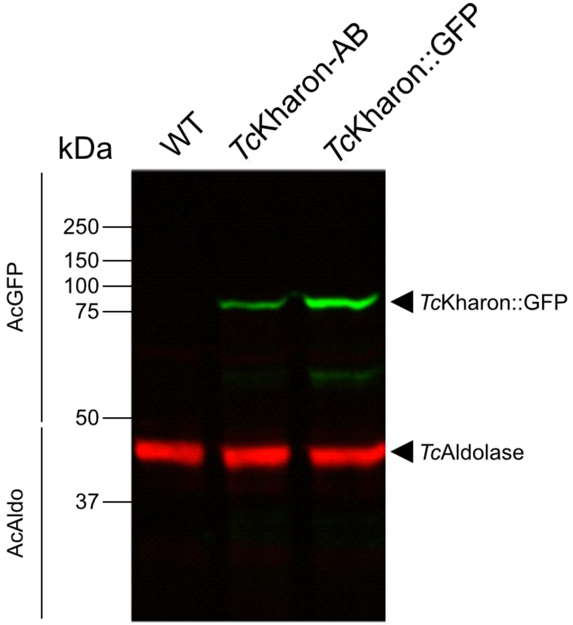

Supplementary Figure S5. Analysis of fluorescence of the *Tc*Kharon-AB cells. A. Flow cytometry chart. Data were analyzed with FlowJo 7.6 version. WT (control) is compared with *Tc*Kharon-AB cells. The histogram on the right indicates positive fluorescent cells (GFP+). **B.** The same cultures were lyzed, run on an SDS-PAGE gel then transferred to a PDVF membrane. The membrane was incubated with an α-GFP primary antibody and an α-rabbit secondary antibody. A band corresponding to the fused protein *Tc*Kharon::GFP is highlighted.
